# High Transmission Rates of Early Omicron Subvariant BA.2 in Bangkok, Thailand

**DOI:** 10.1155/2023/4940767

**Published:** 2023-12-06

**Authors:** Sininat Petcharat, Ananporn Supataragul, Piyapha Hirunpatrawong, Pattama Torvorapanit, Chonticha Klungthong, Piyawan Chinnawirotpisan, Sasiprapa Ninwattana, Nattakarn Thippamom, Leilani Paitoonpong, Gompol Suwanpimolkul, Watsamon Jantarabenjakul, Rome Buathong, Khajohn Joonlasak, Wudtichai Manasatienkij, Khwankamon Rattanatumhi, Napaporn Chantasrisawad, Nuntana Chumpa, Thomas S. Cotrone, Stefan Fernandez, Sira Sriswasdi, Supaporn Wacharapluesadee, Opass Putcharoen

**Affiliations:** ^1^Thai Red Cross Emerging Infectious Diseases Clinical Center, King Chulalongkorn Memorial Hospital, Bangkok 10330, Thailand; ^2^Department of Medicine, Faculty of Medicine, Chulalongkorn University, Bangkok 10330, Thailand; ^3^Division of Infectious Diseases, Department of Medicine, Faculty of Medicine, Chulalongkorn University, Bangkok 10330, Thailand; ^4^Department of Virology, Armed Forces Research Institute of Medical Sciences, Bangkok 10400, Thailand; ^5^Department of Disease Control, Ministry of Public Health, Muang, Nonthaburi 11000, Thailand; ^6^Center of Excellence in Computational Molecular Biology, Faculty of Medicine, Chulalongkorn University, Bangkok 10330, Thailand; ^7^Center for Artificial Intelligence in Medicine, Research Affairs, Faculty of Medicine, Chulalongkorn University, Bangkok 10330, Thailand

## Abstract

The emergence of Omicron as the fifth variant of concern within the SARS-CoV-2 pandemic in late 2021, characterized by its rapid transmission and distinct spike gene mutations, underscored the pressing need for cost-effective and efficient methods to detect viral variants, especially given their evolving nature. This study sought to address this need by assessing the effectiveness of two SARS-CoV-2 variant classification platforms based on RT-PCR and mass spectrometry. The primary aim was to differentiate between Delta, Omicron BA.1, and Omicron BA.2 variants using 618 COVID-19-positive samples collected from Bangkok patients between November 2011 and March 2022. The analysis revealed that both BA.1 and BA.2 variants exhibited significantly higher transmission rates, up to 2-3 times, when compared to the Delta variant. This research presents a cost-efficient approach to virus surveillance, enabling a quantitative evaluation of variant-specific public health implications, crucial for informing and adapting public health strategies.

## 1. Introduction

Omicron emerged as the fifth variant of concern (VOC) of coronavirus disease (COVID-19) in November 2021, replacing the predominant Delta variants. Omicron was first identified on November 11, 2021, in Botswana, and on November 14, 2021, in South Africa [1]. Omicron contains more than 30 mutations on its spike protein, including 15 mutations in the receptor-binding domain (RBD) that might underlie its increased transmissibility and reduced vaccine efficacy [2]. In April 2022, the World Health Organization (WHO) announced the BA.1, BA.2, BA.3, BA.4, and BA.5 Omicron subvariants for surveillance [3]. Hence, the ability to detect these new variants is required to monitor their spread, evaluate their clinical impact, and update public health policy.

The most common lineages of Omicron in early 2022 were BA.1 (B.1.1.529.1), BA.2 (B.1.1.529.2), and BA.3 (B.1.1.529.3). These variants share 12 mutations in the RBD which binds to human angiotensin-converting enzyme 2 (ACE2) proteins and is responsible for viral entry into the host cell [4]. Additionally, these variants also share 21 common mutations in other regions of the spike protein, such as the N501Y and Q498R mutations that are expected to enhance the binding to ACE2 receptors and the H655Y, N679K, and P681H mutations that are believed to increase spike cleavage and facilitate virus transmission [5]. BA.2 is of particular interest because it was reportedly 1.5-fold more infectious than BA.1 and 4.2 times more than Delta. BA.2 has a 30% higher potential than BA.1 to escape existing vaccines and is 17-fold more capable than Delta [6] in this regard. BA.2 is 35-fold more resistant to sotrovimab, a monoclonal antibody, compared to the ancestral D614G-bearing B.1.1 virus. Moreover, BA.2 is 6.4-fold more resistant than BA.1 in neutralization assay using murine sera [7]. BA.2 contains S371F, T376A, D405N, and R408S substitutions in the RBD, which might increase its rate of spread [8], along with unique mutations, T19I, L24S, P25del, P26del, A27S, V213G, T376A, and R408S [4].

The evolutionary rate of SARS-CoV-2 has accelerated due to multiple factors. Immune evasion from vaccination, past infections, and hybrid immunity are the key drivers of this phenomenon [9]. In addition, intrahost evolution in immunocompromised hosts may lead to unexpected mutations and the emergence of novel variants [10–12]. Current rapid mutations in the spike protein of SARS-CoV-2 may also alter the sensitivity and specificity of reverse-transcription polymerase chain reaction. Hence, the designed primers and RT-qPCR assays for detecting SARS-CoV-2 variants need to be constantly updated to capture Omicron sublineages [13].

**Figure 1:**
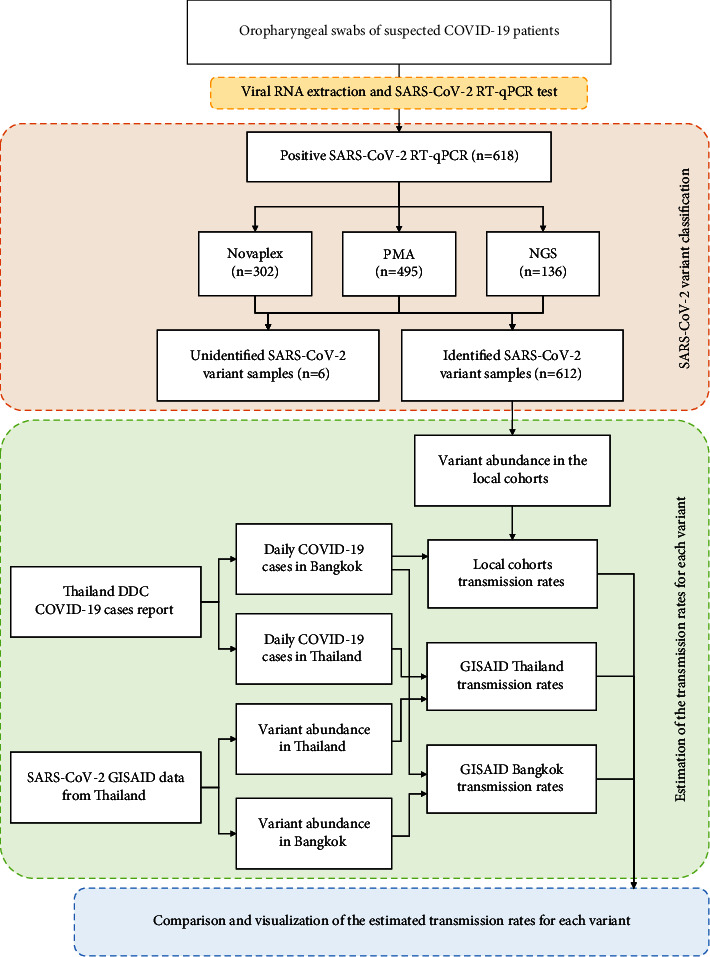
Workflow of the study starting from sample collection to data analysis. The number of samples that underwent the three SARS-CoV-2 variant classification methods is larger than the number of positive SARS-CoV-2 RT-qPCR samples as the variant of some samples was classified by multiple methods.

In Thailand, Omicron is the fifth wave of the COVID-19 pandemic that started around January 2022 and spread much faster than the earlier Delta variants [14]. This situation prompted our team, the Thai Red Cross Emerging Infectious Diseases Clinical Center (TRC-EIDCC), to develop a cost-effective and rapid workflow for classifying SARS-CoV-2 variants in patients who visited the King Chulalongkorn Memorial Hospital (KCMH). In this study, we compared whole-genome sequencing, which is the gold standard method for SARS-CoV-2 variant classification, to more affordable array-based (Novaplex™ SARS-CoV-2 Variants VII) and mass spectrometry-based methods (MassARRAY®). The collected data let us derive an estimate for the increased transmission rate of the Omicron variants compared to the Delta variant that is consistent with estimates obtained from GISAID data [15]. Hence, the ability to detect viral variants using affordable technology can enable a sentinel surveillance site to quantitatively monitor and evaluate the impact of an outbreak. The overall workflow of this study is shown in Figure 1.

## 2. Materials and Methods

### 2.1. Swab Sampling and Viral RNA Extraction

Oropharyngeal swabs of suspected COVID-19 patients were collected between 5 November 2021 and 31 March 2022 as a part of routine SARS-CoV-2 surveillance at the TRC-EIDCC from KCMH (IRB = No. 361/59, 400/63), Suvarnabhumi Airport (Division of International Communicable Disease Control Port and Quarantine) and other organizations in Bangkok. Viral RNA was extracted from the samples using a MagPurix® 12 EVO Automated Nucleic Acid Purification System (Zinext Life Science Corp) and confirmed for SARS-CoV-2 by reverse-transcription PCR (RT-qPCR) test.

### 2.2. SARS-CoV-2 Variant Classification

As mentioned, three methods were used to classify SARS-CoV-2 variants in positive samples, namely, Novaplex™ SARS-CoV-2 Variants VII Assay (Novaplex) (Seegene Technologies), MassARRAY® System (Agena Bioscience), and whole genome sequencing (WGS) using next-generation sequencing (NGS) (Illumina). Ct values generated by the RT-qPCR test were considered together with the urgency of the samples to determine the variant classification method(s) used for each sample.  Table 1 compares the performance of the three assays. The samples with discordant variant results were subjected to multiple assays for confirmation. For discordant results, more weights are given to assays with higher specificity (NGS followed by MassARRAY® and Novaplex™).

For Novaplex, the detection of E484A and HV69/70 deletion in spike gene, N501Y in RdRP gene, and endogenous internal control were performed according to the manufacturer's instructions on the CFX96 Touch Real-Time PCR Detection System (Bio-Rad, Hercules, CA). The test results were analyzed with Seegene software using a positive cut-off of Ct < 42. The list of targeted mutations is provided in Table 2.

For the MassARRAY® System, a multiplex PCR MassARRAY assay (PMA) was conducted using specific point mutation panels. Four different point mutation panels of PMA were designed based on the circulating variants and used as the assay throughout the period, namely, ABDO V1, Omicron V1, Omicron V2, and Omicron V3 (Table 2). Samples with Ct < 30 were analyzed with RT-PCR using iPLEX prochemistry reagent for target regions amplification and MALDI-TOF mass spectrometer (MassARRAY Analyzer) [16] to detect nucleotide at target mutations of each panel.

For WGS, viral RNA was amplified by ARTIC V3 and V4 protocols. The DNA library was prepared using an Illumina® DNA Prep kit with Respiratory Virus Oligos Panel v2 (Illumina) enrichment. Sequencing was performed on a MiSeq platform using a 2 × 250 nucleotides reagent kit v2 and assembled by mapping with the reference genome Wuhan-Hu-1 (NC_045512.2) as previously described in [17]. A variant of the genomes was classified using Pangolin [18] and Nextclade [19].

### 2.3. Estimation of the Transmission Rates for Each Variant

The number of new cases at time *t* + 1, *N*_*t*+1_, was modeled using three factors, the current number of cases, *N*_*t*_, the current fractional abundance of each variant, {*f*_*t*_^Delta^, *f*_*t*_^BA.1^, *f*_*t*_^BA.2^}, and the transmission rate of each variant {*r*^Delta^, *r*^BA.1^, *r*^BA.2^}, which represents the number of new cases that could arise from an infected person over a period of time and is assumed to be time-independent:

(1) $$\begin{array}{c} N_{t+1}=N_{t}\underset{v\in \left\{\text{Delta},\text{Omicron},\text{BA}.2\right\}}{\sum }f_{t}^{v}r^{v}. \end{array}$$

Here, a unit of time was set at 5 days. A first-order competition model was used to estimate the dynamics of the fractional abundance of viral variants:

(2) $$\begin{array}{c} f_{t+1}^{s}=\frac{f_{t}^{s}r^{s}}{\sum_{v\in \left\{\text{Delta},\text{Omicron},\text{BA}.2\right\}}f_{t}^{v}r^{v}}. \end{array}$$

The search for the best-fitted transmission rate of each variant {*r*^Delta^, *r*^BA.1^, *r*^BA.2^} was performed using SciPy's minimize function with weighted mean squared error (weighted by the number of tested samples at each time point) as the objective. To estimate the variability of the fitted transmission rates, the parameter fitting process was repeated on 100 random initial guesses for the transmission rates, each drawn uniformly from [0, 1] and 100 bootstrap sampling of the time-series daily case data, each drawn from two-third of the number of time points without replacement. The number of new cases in Bangkok during the time period was collected from the Thailand Ministry of Public Health record. The fractional abundances of the Delta, BA.1, and BA.2 variants in Bangkok during the time period were estimated based on either our local samples or submitted entries on GISAID.

## 3. Results

**Figure 2:**
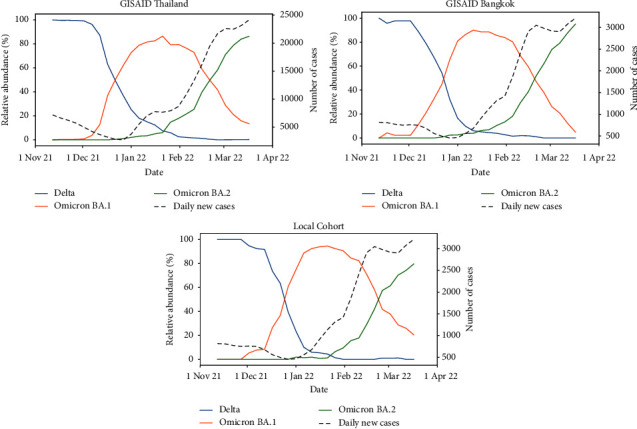
The trends of daily new cases and relative abundances of the Delta and Omicron variants from November 2021 to March 2022 in Thailand, Bangkok, and our hospital. Variant abundance data for Thailand and Bangkok were retrieved from GISAID. Numbers of daily new cases were retrieved from the report released by the Thailand Ministry of Public Health. Data were smoothed with a 5-day average sliding window.

The TRC-EIDCC identified the first Omicron case (BA.1) from a sample from Suvarnabhumi airport on 8 December 2021, when the number of daily new cases in Thailand was around 3,000–4,000 cases. Then, the first Omicron BA.2 case was detected on 8 January 2022, when the number of daily new cases reached 10,000. As shown in Figure 2, the new Omicron variants quickly replaced the prevalent Delta variant in early January, although some Delta cases can still be found up until early March. The BA.2 lineage then replaced BA.1 as the most dominant lineage in early March. Similar relative abundances of the three variants of interest, Delta, Omicron BA.1, and Omicron BA.2, were obtained with either GISAID data (*n* = 4,295 for Bangkok and *n* = 11,422 for Thailand) or our cohorts (*n* = 612).

From 5 November 2021 to 31 March 2022 (21 weeks), a total of 618 samples tested positive for SARS-CoV-2 were analyzed at our center using three assays, namely, Novaplex, PMA, and WGS, to identify SARS-CoV-2 variants. As the resolutions of the three assays are different, the subvariants detected by PMA and NGS were grouped with the parent subvariants that are detectable by Novaplex, namely, Delta, Omicron BA.1, and Omicron BA.2. Out of 618 samples, 261 were subjected to multiple assays, and only nine were discordant (Table 3). All discordant results were due to Novaplex's limited ability to detect mutations. The variants of 6 samples were unidentified as they failed the assays or yielded inconclusive results. Hence, only the variants of 612 samples were considered in the downstream analysis.

**Figure 3:**
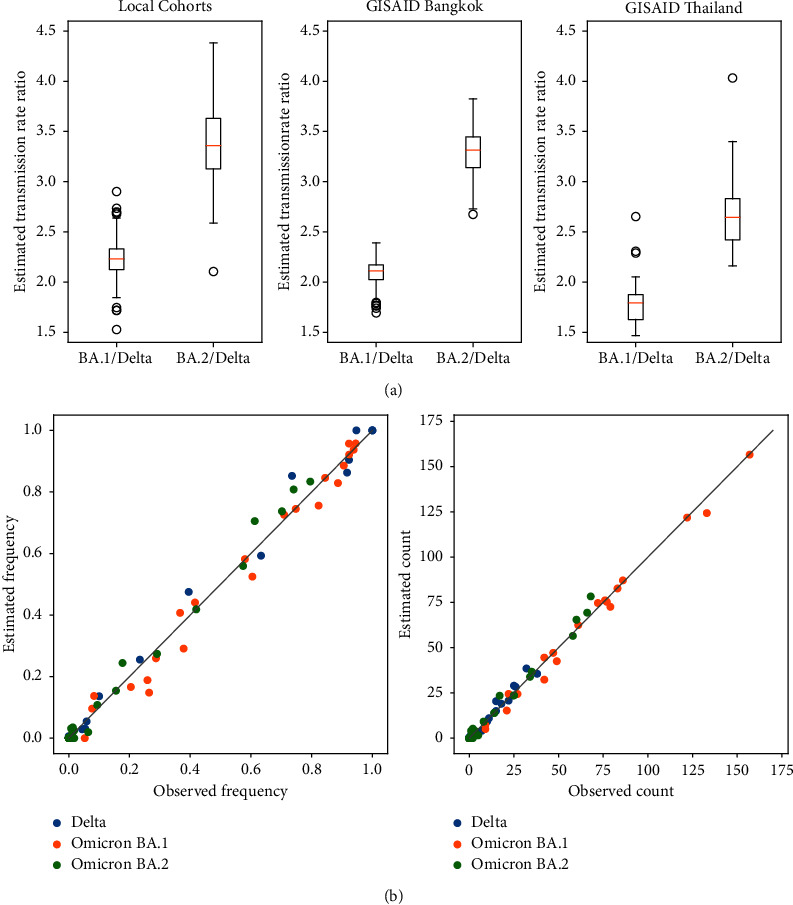
Estimated transmission rate ratios for Omicron BA.1 and BA.2 variants relative to the Delta variant (a) and goodness of fit between estimated transmission rates and observations (b). (a) Variant abundance data from local cohorts, GISAID entries for Bangkok, and GISAID entries for Thailand were used. The distributions of the rate ratios were estimated from 100 optimization repeats with different randomly initialized values (see Methods). Mean values were denoted by orange bars. Boxes indicate the interquartile ranges. Whiskers indicate the 1.5x ranges below the first and above the third quartiles. Circles denote outliers. (b) Scatter plots show the agreement between observed variant frequencies and case counts versus the predictions based on estimated transmission rates. Each data point corresponds to a 5-day period from November 2021 to March 2022. Results for the estimated transmission rates with the lowest mean square error on data from our hospital are shown.

To estimate the transmission rates, i.e., the number of new infections that could arise on average from an infected individual, a linear model linking the relative abundance and the transmission rate of each variant to the number of daily cases was built (see Methods). The estimation process was repeated 100 times with different random initial guesses to determine the uncertainty. As shown in Figure 3(a), despite small sample counts, data from our local cohorts (*n* = 612) yielded a similar estimate as Bangkok data from GISAID (*n* = 4,295). The transmission rate for Omicron BA.1 was estimated to be 2.23 (SD = 0.22) and 2.09 (SD = 0.14) times that of the Delta variant, while the transmission rate for Omicron BA.2 was estimated to be 3.38 (SD = 0.43) and 3.29 (SD = 0.24) times that of the Delta variant. Interestingly, using Thailand data from GISAID yielded significantly lower estimates of 1.78 (SD = 0.18) for BA.1 relative to Delta and 2.67 (SD = 0.38) for BA.2 relative to Delta, respectively (Mann–Whitney *U* test *p* values <3*e* − 24). The baseline transmission rate for the Delta variant was estimated to be 0.58 (SD = 0.06), 0.59 (SD = 0.04), and 0.66 (SD = 0.06) using local data, GISAID data for Bangkok, and GISAID data for Thailand, respectively. In all cases, these estimates fit well with the observed abundances and case counts (Figure 3(b)). The lower transmission rates estimated using data from all over Thailand compared to Bangkok data fit the expectation that higher transmissibility would be observed in densely populated areas, like Bangkok, compared to more rural areas.

## 4. Discussion

The Omicron BA.1 variant (B.1.1.529) rapidly replaced the predominant Delta strain within 4 weeks, leading to the fifth wave of COVID-19 in Thailand (Figure 2). The rapid spread of Omicron was similar across countries; however, the immunity from infection and vaccination differed, such as the cases in Denmark [8], South Africa [20], and EU [21]. Differences in mutations on the spike protein of Omicron BA.1 and BA.2 may explain their high transmissibility. BA.2 has deletions at amino acid positions 24–26 and A27S substitution, whereas BA.1 has deletions at amino acid positions 69-70 and 142–144. These positions are located near the N-terminal domain (NTD) antigenic site and are associated with resistance to neutralizing monoclonal antibodies [22]. The deletion at amino acid position 69-70 in spike protein affects the antigenicity leading to resistance against neutralizing antibodies and defines the sublineages BA.1 [23].

The Novaplex™ assay is easy to use, fast, cost-effective, and able to handle low-concentration samples (Ct < 42). However, this assay can detect only three point mutations, E484A, HV69/70 deletion, and N501Y, which are insufficient for distinguishing other subvariants of Delta and Omicron. On the other hand, the PMA platform can accommodate up to 40 point mutations, producing more information for classifying subvariants. Furthermore, PMA utilizes PCR and mass spectrometry which are not as expensive as WGS and is applicable to samples with lower viral loads (Ct < 35 compared to Ct < 26 for WGS). [16] Although WGS is still a gold standard method for variant classification and novel variant identification, PMA and Novaplex™ can be beneficial for screening variants in the high transmission areas and for preselecting samples for WGS. In particular, the choices of 40-point mutations in PMA can be continually updated to encompass new variants, as done in this study (Table 2). These assays are also highly concordant (96.5%, 252 out of 261 cases).

A key highlight of our study is that data from a sentinel site with a limited number of samples (*n* = 612) can still faithfully reflect the variant abundance profiles and the transmission rates compared to those obtained using the much larger provincial level and national level datasets from GISAID (Figures 3(a) and 3(b)). This stresses the importance of capacity building in basic viral genomics and mathematical modeling at sentinel hospitals which would enable them to quantitatively assess outbreak situations and inform public health policy. However, the lack of epidemiological data means that our modeling can only capture the average transmission characteristics of the virus. Information on the actual transmission rates in the community was also unavailable for validating our estimates. Furthermore, it should be noted that external factors, such as the saturation of PCR testing capacity, underreporting of new cases, and changes in public health policy, can confound the observations. These details are needed to fully ascertain the accuracy of our approach.

In addition to through patients, temporal changes in SARS-CoV-2 evolution and variant compositions can also be effectively monitored in environmental samples, such as wastewater [24]. While hospital surveillance captures linkage across viral variants, clinical severity, and human-to-human transmission, environment-based surveillance can illustrate a more complete picture of the reservoir of viral variants in a community and supplement transmission route reconstruction. However, array-based variant detection approaches utilized here will have limited application for environment-based surveillance because they cannot fully deconvolute the mixture of variants within the samples.

## 5. Conclusion

The use of the affordable mass spectrometry-based MassARRAY® System for detecting SARS-CoV-2 variants in clinical samples enabled sentinel surveillance at a primary healthcare institution. This method is also flexible, allowing primer customization to target new emerging mutations, and has a rapid turnaround time. The ability to monitor and predict the current magnitude of infection and change in transmission rate using our strategy facilitates prompt allocation of vaccines and treatment resources that prevents overburden of hospital admission.

## Figures and Tables

**Table 1:** SARS-CoV-2 variant classification assays comparison.

Performance	Novaplex	PMA	NGS
Detected point mutations	3 specific point mutations to identify 3 variants	Up to 40 customizable point mutations to identify >3 variants	All point mutations across the whole genome
Turnaround time	3–5 hours	1-2 days	2-3 days
Limitation Ct	<42	<35	<26
Estimated cost	$57 per sample	$57 per sample	$180 per sample
Scope of the assay	Known variant surveillance Rapid screening	Known variant surveillance Coinfection from different variants	Known and novel variant identification

**Table 2:** Mutations targeted by Novaplex™ SARS-CoV-2 variants VII assay and three versions of PMA MassARRAY panels.

Method	Mutation	Control
Novaplex™ SARS-CoV-2 variants VII assay	S gene: HV69del, E484A, N501Y	RdRp gene

MassARRAY® ABDO V1^a^	S gene: S13I, T20N, A67V, HV69del, D80A, Y144del, W152C, F157L, R190S, LLA241del, D253G, V367F, K417N, K417T, L452R, E484Q, E484K, N501Y, A570D, Q613H, D614G, H655Y, Q677H, P681R, P681H, A701V, T716I, F888L, S982A, T1027I, E1092K, H1101Y, V1176F, D1118H	N gene: C29208del
MassARRAY® Omicron V1^a^	S gene: T19R, T19I, A67V, HV69del, T95I, GVY142del, N211del, G339D, S373P, S375F, K417N, S477N, T478K, E484A, Q493R, Q498R, N501Y, Y505H, T574K, D614G, H655Y, P681R, P681H, D796Y, N856K, Q954H, N969K, L981F, D1118H	
MassARRAY® Omicron V2^a^	S gene: T19R, T19I, A67V, HV69del, T95I, GVY142del, N211del, G339D, R346K, S373P, S375F, K417N, S477N, T478K, E484A, Q493R, Q498R, N501Y, Y505H, T547K, D614G, H655Y, P681R, P681H, D796Y, N856K, Q954H, N969K, L981F, D1118H, I1221T	
MassARRAY® Omicron V3^a^	S gene: V3G, T19I, T19R, A67V, HV69del, T95I, G142D, K147E, G339H, G339D, R346K, K417N, L452R, N460K, S477N, T478K, E484A, F486V, Q493R, Q498R, N501Y, Y505H, T547K, D614G, H655Y, P681H, P681R, D796Y, N856K, Q954H, L981F, I1221T	
	N gene: P151S, ORF7b: L11F	

^a^Different mutation panels were used at different time periods as the assay was continually improved. ABDO V1 was used until 16 December 2021. Omicron V1 was used from 18 December 2021 until 3 January 2022. Omicron V2 was used from 4 January 2022 until 28 March 2022. Omicron V3 was used from 27 March 2022 onwards.

**Table 3:** Number of positive samples detected by each assay combination.

Novaplex	PMA	WGS	Samples	Mismatches^b^
x			68	
	x		279	
		x	4	
x	x		137	8
x		x	48	1
	x	x	35	—
x	x	x	41	—
			6^a^	
Total			618	9

^a^Samples that failed the variant classification step, two samples from each method. ^b^Mismatch results, different variants detected by multiple methods, were cleaned before data analysis by basing the result on the more reliable method, ranging from the gold standard WGS, PMA, and Novaplex.

## Data Availability

SARS-CoV-2 whole-genome sequences generated in this study are deposited into the GISAID repository (https://www.gisaid.org). GISAID ID and the SARS-CoV-2 variant classification results of 618 positive SARS-CoV-2 samples analyzed by three methods are provided in Supplementary Table S1. New daily SARS-CoV-2 cases in Bangkok and Thailand were retrieved from the Thailand Department of Disease Control COVID-19 API (https://ddc.moph.go.th/covid19-daily-dashboard/); the data are also provided in Supplementary Table S2.

## Supplementary Materials

Supplementary Materials Supplementary Table S1: SARS-CoV-2 variant classification results by Novaplex™ SARS-CoV-2 Variants VII Assay, MassARRAY®, and Next-Generation Sequencing (NGS). Results summary is concluded from the three methods with more weights given to NGS over MassARRAY® and Novaplex™ for unmatched results. Supplementary Table S2: new daily SARS-CoV-2 cases in Bangkok and Thailand were retrieved from the Thailand Department of Disease Control COVID-19 API (https://ddc.moph.go.th/covid19-daily-dashboard/).
