# Supplementary material for: High Transmission Rates of Early Omicron Subvariant BA.2 in Bangkok, Thailand
Source: Adv Virol. 2023 Dec 6;2023:4940767. doi: 10.1155/2023/4940767 (PMC10719011; doi:10.1155/2023/4940767)
Supplement: Supplementary Materials — Supplementary Table S1: SARS-CoV-2 variant classification results by Novaplex™ SARS-CoV-2 Variants VII Assay, MassARRAY®, and Next-Generation Sequencing (NGS). Results summary is concluded from the three methods with more weights given to NGS over MassARRAY® and Novaplex™ for unmatched results. Supplementary Table S2: new daily SARS-CoV-2 cases in Bangkok and Thailand were retrieved from the Thailand Department of Disease Control COVID-19 API (https://ddc.moph.go.th/covid19-daily-dashboard/). [file 4940767.f1.docx]

Table S1. SARS-CoV-2 variant classification results by Novaplex™ SARS-CoV-2 Variants VII Assay, MassARRAY® and Next-Generation Sequencing (NGS). Results summary are concluded from the three methods with weight given to NGS > MassARRAY® > Novaplex™ for unmatched results.

| **Sample no.** | **Collection date** | **Results summary** | **Match** | **Novaplex™ results** | **MassARRAY panels** | **MassARRAY® results** | **NGS results** | **GISAID ID** |
| --- | --- | --- | --- | --- | --- | --- | --- | --- |
| 1 | 05-Nov-21 | Delta |  |  |  |  | Delta (AY.30) | EPI_ISL_15592951 |
| 2 | 05-Nov-21 | Delta |  |  |  |  | Delta (AY.85) | EPI_ISL_15592952 |
| 3 | 16-Nov-21 | Delta |  |  | ABDO V1 panel | Delta (B.1.617.2) |  |  |
| 4 | 16-Nov-21 | Delta |  |  | ABDO V1 panel | Delta (B.1.617.2) |  |  |
| 5 | 16-Nov-21 | Delta |  |  | ABDO V1 panel | Delta (B.1.617.2) |  |  |
| 6 | 16-Nov-21 | Delta |  |  | ABDO V1 panel | Delta (B.1.617.2) |  |  |
| 7 | 16-Nov-21 | Delta |  |  | ABDO V1 panel | Delta (B.1.617.2) |  |  |
| 8 | 16-Nov-21 | Delta | Yes |  | ABDO V1 panel | Delta (B.1.617.2) | Delta (AY.30) | EPI_ISL_7131983 |
| 9 | 16-Nov-21 | Delta | Yes |  | ABDO V1 panel | Delta (B.1.617.2) | Delta (AY.30) | EPI_ISL_7131984 |
| 10 | 18-Nov-21 | Delta | Yes |  | ABDO V1 panel | Delta (B.1.617.2) | Delta (AY.85) | EPI_ISL_7131985 |
| 11 | 19-Nov-21 | Delta |  |  | ABDO V1 panel | Delta (B.1.617.2) |  |  |
| 12 | 19-Nov-21 | Delta | Yes |  | ABDO V1 panel | Delta (B.1.617.2) | Delta (B.1.617.2) | EPI_ISL_7131986 |
| 13 | 19-Nov-21 | Delta |  |  | ABDO V1 panel | Delta (B.1.617.2) |  |  |
| 14 | 29-Nov-21 | Delta |  |  | ABDO V1 panel | Delta (B.1.617.2) |  |  |
| 15 | 29-Nov-21 | Delta |  |  | ABDO V1 panel | Delta (B.1.617.2) |  |  |
| 16 | 29-Nov-21 | Delta |  |  | ABDO V1 panel | Delta (B.1.617.2) |  |  |
| 17 | 30-Nov-21 | Delta |  |  | ABDO V1 panel | Delta (B.1.617.2) |  |  |
| 18 | 01-Dec-21 | Delta |  |  | ABDO V1 panel | Delta (B.1.617.2) |  |  |
| 19 | 01-Dec-21 | Delta |  |  | ABDO V1 panel | Delta (B.1.617.2) |  |  |
| 20 | 01-Dec-21 | Delta |  |  | ABDO V1 panel | Delta (B.1.617.2) |  |  |
| 21 | 01-Dec-21 | Delta |  |  | ABDO V1 panel | Delta (B.1.617.2) |  |  |
| 22 | 03-Dec-21 | Delta |  |  | ABDO V1 panel | Delta (B.1.617.2) |  |  |
| 23 | 02-Dec-21 | Delta |  |  | ABDO V1 panel | Delta (B.1.617.2) |  |  |
| 24 | 02-Dec-21 | Delta |  |  | ABDO V1 panel | Delta (B.1.617.2) |  |  |
| 25 | 02-Dec-21 | Delta |  |  | ABDO V1 panel | Delta (B.1.617.2) |  |  |
| 26 | 01-Dec-21 | Delta |  |  | ABDO V1 panel | Delta (B.1.617.2) |  |  |
| 27 | 03-Dec-21 | Delta |  |  | ABDO V1 panel | Delta (B.1.617.2) |  |  |
| 28 | 07-Dec-21 | Delta |  |  | ABDO V1 panel | Delta (B.1.617.2) |  |  |
| 29 | 07-Dec-21 | Delta |  |  | ABDO V1 panel | Delta (B.1.617.2) |  |  |
| 30 | 07-Dec-21 | Failed |  |  | ABDO V1 panel | Unidentified |  |  |
| 31 | 07-Dec-21 | Failed |  |  | ABDO V1 panel | Unidentified |  |  |
| 32 | 09-Dec-21 | Delta |  |  | ABDO V1 panel | Delta (B.1.617.2) |  |  |
| 33 | 08-Dec-21 | Delta |  |  | ABDO V1 panel | Delta (B.1.617.2) |  |  |
| 34 | 08-Dec-21 | Omicron BA.1 | Yes | Possible Omicron (B.1.1.529) | ABDO V1 panel | Omicron (BA.1) |  |  |
| 35 | 13-Dec-21 | Delta |  | Unidentified | ABDO V1 panel | Delta (B.1.617.2) |  |  |
| 36 | 13-Dec-21 | Delta |  |  | ABDO V1 panel | Delta (B.1.617.2) |  |  |
| 37 | 14-Dec-21 | Delta | Yes |  | ABDO V1 panel | Delta (B.1.617.2) | Delta (AY.30) | EPI_ISL_11512481 |
| 38 | 14-Dec-21 | Delta | Yes |  | ABDO V1 panel | Delta (B.1.617.2) | Delta (AY.85) | EPI_ISL_11512482 |
| 39 | 14-Dec-21 | Delta | Yes |  | ABDO V1 panel | Delta (B.1.617.2) | Delta (AY.85) | EPI_ISL_11512483 |
| 40 | 14-Dec-21 | Delta | Yes | Non-omicron/ possible Delta | ABDO V1 panel | Delta (B.1.617.2) | Delta (AY.85) | EPI_ISL_11512484 |
| 41 | 15-Dec-21 | Delta | Yes | Non-omicron/ possible Delta |  |  | Delta (AY.85) | EPI_ISL_11512485 |
| 42 | 15-Dec-21 | Delta | Yes | Non-omicron/ possible Delta | ABDO V1 panel | Delta (B.1.617.2) | Delta (AY.85) | EPI_ISL_11512486 |
| 43 | 15-Dec-21 | Delta | Yes | Non-omicron/ possible Delta |  |  | Delta (AY.85) | EPI_ISL_11512487 |
| 44 | 15-Dec-21 | Omicron BA.1 | Yes | Possible Omicron (B.1.1.529) | ABDO V1 panel | Omicron (BA.1) |  |  |
| 45 | 16-Dec-21 | Delta | Yes |  | ABDO V1 panel | Delta (B.1.617.2) | Delta (AY.85) | EPI_ISL_11512488 |
| 46 | 16-Dec-21 | Delta | Yes |  | ABDO V1 panel | Delta (B.1.617.2) | Delta (AY.85) | EPI_ISL_11512489 |
| 47 | 17-Dec-21 | Delta | Yes | Non-omicron/ possible Delta | Omicron V1 panel | Delta (B.1.617.2) |  |  |
| 48 | 18-Dec-21 | Delta | Yes | Non-omicron/ possible Delta | Omicron V1 panel | Delta (B.1.617.2) | Delta (AY.85) | EPI_ISL_11512490 |
| 49 | 20-Dec-21 | Delta | Yes | Non-omicron/ possible Delta |  |  | Delta (AY.85) | EPI_ISL_11512491 |
| 50 | 20-Dec-21 | Delta | Yes | Non-omicron/ possible Delta | Omicron V1 panel | Delta (B.1.617.2) |  |  |
| 51 | 20-Dec-21 | Delta | Yes | Non-omicron/ possible Delta |  |  | Delta (AY.85) | EPI_ISL_11512492 |
| 52 | 20-Dec-21 | Delta | Yes | Non-omicron/ possible Delta | Omicron V1 panel | Delta (B.1.617.2) |  |  |
| 53 | 20-Dec-21 | Delta |  |  |  |  | Delta (AY.30) | EPI_ISL_17623599 |
| 54 | 22-Dec-21 | Delta | Yes | Non-omicron/ possible Delta |  |  | Delta (AY.85) | EPI_ISL_11512493 |
| 55 | 21-Dec-21 | Omicron BA.1 | Yes | Possible Omicron (B.1.1.529) | Omicron V1 panel | Omicron (BA.1) | Omicron (BA.1) | EPI_ISL_10894108 |
| 56 | 21-Dec-21 | Omicron BA.1 | Yes |  | Omicron V1 panel | Omicron (BA.1) | Omicron (BA.1) | EPI_ISL_10894109 |
| 57 | 21-Dec-21 | Omicron BA.1 | Yes |  | Omicron V1 panel | Omicron (BA.1) | Omicron (BA.1) | EPI_ISL_10894110 |
| 58 | 21-Dec-21 | Omicron BA.1 |  | Unidentified | Omicron V1 panel | Omicron (BA.1) |  |  |
| 59 | 21-Dec-21 | Omicron BA.1 | Yes |  | Omicron V1 panel | Omicron (BA.1) | Omicron (BA.1) | EPI_ISL_10894111 |
| 60 | 22-Dec-21 | Delta | Yes | Non-omicron/ possible Delta | Omicron V1 panel | Delta (B.1.617.2) |  |  |
| 61 | 22-Dec-21 | Delta | Yes | Non-omicron/ possible Delta | Omicron V1 panel | Delta (B.1.617.2) |  |  |
| 62 | 22-Dec-21 | Delta | Yes | Non-omicron/ possible Delta | Omicron V1 panel | Delta (B.1.617.2) |  |  |
| 63 | 23-Dec-21 | Delta | Yes | Non-omicron/ possible Delta |  |  | Delta (AY.85) | EPI_ISL_11512495 |
| 64 | 24-Dec-21 | Omicron BA.1 |  |  | Omicron V1 panel | Omicron (BA.1) |  |  |
| 65 | 22-Dec-21 | Omicron BA.1 |  |  | Omicron V1 panel | Omicron (BA.1) |  |  |
| 66 | 25-Dec-21 | Omicron BA.1 |  |  | Omicron V1 panel | Omicron (BA.1) |  |  |
| 67 | 25-Dec-21 | Delta | Yes | Non-omicron/ possible Delta |  |  | Delta (AY.85) | EPI_ISL_11512496 |
| 68 | 25-Dec-21 | Delta | Yes | Non-omicron/ possible Delta |  |  | Delta (AY.85) | EPI_ISL_11512497 |
| 69 | 27-Dec-21 | Omicron BA.1 | Yes | Possible Omicron (B.1.1.529) | Omicron V1 panel | Omicron (BA.1) |  |  |
| 70 | 27-Dec-21 | Delta | Yes | Non-omicron/ possible Delta |  |  | Delta (AY.85) | EPI_ISL_11512498 |
| 71 | 27-Dec-21 | Delta | Yes | Non-omicron/ possible Delta |  |  | Delta (AY.85) | EPI_ISL_11512499 |
| 72 | 27-Dec-21 | Delta | Yes | Non-omicron/ possible Delta | Omicron V1 panel | Delta (B.1.617.2) |  |  |
| 73 | 27-Dec-21 | Delta | Yes | Non-omicron/ possible Delta | Omicron V1 panel | Delta (B.1.617.2) |  |  |
| 74 | 28-Dec-21 | Delta | Yes | Non-omicron/ possible Delta | Omicron V1 panel | Delta (B.1.617.2) | Delta (AY.30) | EPI_ISL_11563561 |
| 75 | 27-Dec-21 | Omicron BA.1 | Yes |  | Omicron V1 panel | Omicron (BA.1) | Omicron (BA.1) | EPI_ISL_10894112 |
| 76 | 27-Dec-21 | Omicron BA.1 |  |  | Omicron V1 panel | Omicron (BA.1) |  |  |
| 77 | 27-Dec-21 | Omicron BA.1 |  |  | Omicron V1 panel | Omicron (BA.1) |  |  |
| 78 | 27-Dec-21 | Omicron BA.1 |  |  | Omicron V1 panel | Omicron (BA.1) |  |  |
| 79 | 27-Dec-21 | Omicron BA.1 |  |  | Omicron V1 panel | Omicron (BA.1) |  |  |
| 80 | 28-Dec-21 | Delta | Yes | Non-omicron/ possible Delta |  |  | Delta (AY.85) | EPI_ISL_11512500 |
| 81 | 28-Dec-21 | Delta | Yes | Non-omicron/ possible Delta |  |  | Delta (AY.79) | EPI_ISL_11512501 |
| 82 | 28-Dec-21 | Delta | Yes | Non-omicron/ possible Delta | Omicron V1 panel | Delta (B.1.617.2) |  |  |
| 83 | 29-Dec-21 | Omicron BA.1 | Yes | Possible Omicron (B.1.1.529) | Omicron V1 panel | Omicron (BA.1) | Omicron (BA.1) | EPI_ISL_10894113 |
| 84 | 29-Dec-21 | Omicron BA.1 |  |  | Omicron V1 panel | Omicron (BA.1) |  |  |
| 85 | 29-Dec-21 | Delta | Yes | Non-omicron/ possible Delta |  |  | Delta (AY.85) | EPI_ISL_11512502 |
| 86 | 29-Dec-21 | Delta | Yes | Non-omicron/ possible Delta | Omicron V1 panel | Delta (B.1.617.2) |  |  |
| 87 | 29-Dec-21 | Delta | Yes | Non-omicron/ possible Delta |  |  | Delta (AY.85) | EPI_ISL_11512503 |
| 88 | 29-Dec-21 | Delta | Yes | Non-omicron/ possible Delta |  |  | Delta (AY.85) | EPI_ISL_11512504 |
| 89 | 29-Dec-21 | Omicron BA.1 | Yes | Possible Omicron (B.1.1.529) | Omicron V1 panel | Omicron (BA.1) | Omicron (BA.1) | EPI_ISL_10894114 |
| 90 | 29-Dec-21 | Delta | Yes | Non-omicron/ possible Delta | Omicron V1 panel | Delta (B.1.617.2) |  |  |
| 91 | 29-Dec-21 | Omicron BA.1 | Yes | Possible Omicron (B.1.1.529) | Omicron V1 panel | Omicron (BA.1) |  |  |
| 92 | 29-Dec-21 | Omicron BA.1 | Yes | Possible Omicron (B.1.1.529) | Omicron V1 panel | Omicron (BA.1) | Omicron (BA.1) | EPI_ISL_10894115 |
| 93 | 29-Dec-21 | Omicron BA.1 | Yes | Possible Omicron (B.1.1.529) | Omicron V1 panel | Omicron (BA.1) | Omicron (BA.1) | EPI_ISL_10894116 |
| 94 | 30-Dec-21 | Delta | Yes | Non-omicron/ possible Delta | Omicron V1 panel | Delta (B.1.617.2) |  |  |
| 95 | 30-Dec-21 | Omicron BA.1 | Yes | Possible Omicron (B.1.1.529) | Omicron V1 panel | Omicron (BA.1) | Omicron (BA.1) | EPI_ISL_10894117 |
| 96 | 30-Dec-21 | Delta | Yes | Non-omicron/ possible Delta |  |  | Delta (AY.85) | EPI_ISL_11512505 |
| 97 | 30-Dec-21 | Delta | Yes | Non-omicron/ possible Delta |  |  | Delta (AY.79) | EPI_ISL_11512506 |
| 98 | 30-Dec-21 | Delta | Yes | Non-omicron/ possible Delta |  |  | Delta (AY.30) | EPI_ISL_11512507 |
| 99 | 30-Dec-21 | Delta | Yes | Non-omicron/ possible Delta |  |  | Delta (AY.85) | EPI_ISL_11512508 |
| 100 | 30-Dec-21 | Delta | Yes | Non-omicron/ possible Delta |  |  | Delta (AY.30) | EPI_ISL_11512509 |
| 101 | 31-Dec-21 | Delta |  |  | Omicron V1 panel | Delta (B.1.617.2) |  |  |
| 102 | 02-Jan-22 | Omicron BA.1 |  |  | Omicron V1 panel | Omicron (BA.1) |  |  |
| 103 | 02-Jan-22 | Omicron BA.1 |  |  | Omicron V1 panel | Omicron (BA.1) |  |  |
| 104 | 03-Jan-22 | Omicron BA.1 |  |  | Omicron V1 panel | Omicron (BA.1) |  |  |
| 105 | 03-Jan-22 | Omicron BA.1 | Yes | Possible Omicron (B.1.1.529) | Omicron V1 panel | Omicron (BA.1) | Omicron (BA.1.1) | EPI_ISL_11512510 |
| 106 | 03-Jan-22 | Omicron BA.1 | Yes | Possible Omicron (B.1.1.529) | Omicron V1 panel | Omicron (BA.1) | Omicron (BA.1.1) | EPI_ISL_11512511 |
| 107 | 03-Jan-22 | Omicron BA.1 | Yes | Possible Omicron (B.1.1.529) | Omicron V1 panel | Omicron (BA.1) | Omicron (BA.1.1) | EPI_ISL_11512512 |
| 108 | 03-Jan-22 | Omicron BA.1 | Yes | Possible Omicron (B.1.1.529) | Omicron V1 panel | Omicron (BA.1) | Omicron (BA.1) | EPI_ISL_11512513 |
| 109 | 03-Jan-22 | Omicron BA.1 | Yes | Possible Omicron (B.1.1.529) | Omicron V1 panel | Omicron (BA.1) |  |  |
| 110 | 03-Jan-22 | Omicron BA.1 | Yes | Possible Omicron (B.1.1.529) | Omicron V1 panel | Omicron (BA.1) | Omicron (BA.1.1) | EPI_ISL_11512514 |
| 111 | 03-Jan-22 | Omicron BA.1 | Yes | Possible Omicron (B.1.1.529) | Omicron V1 panel | Omicron (BA.1) | Omicron (BA.1) | EPI_ISL_11512515 |
| 112 | 03-Jan-22 | Omicron BA.1 | Yes | Possible Omicron (B.1.1.529) | Omicron V1 panel | Omicron (BA.1) | Omicron (BA.1) | EPI_ISL_11512516 |
| 113 | 03-Jan-22 | Omicron BA.1 | Yes | Possible Omicron (B.1.1.529) | Omicron V1 panel | Omicron (BA.1) | Omicron (BA.1.1) | EPI_ISL_11512517 |
| 114 | 04-Jan-22 | Omicron BA.1 | Yes | Possible Omicron (B.1.1.529) |  |  | Omicron (BA.1) | EPI_ISL_11512518 |
| 115 | 04-Jan-22 | Omicron BA.1 | Yes | Possible Omicron (B.1.1.529) |  |  | Omicron (BA.1.1) | EPI_ISL_11512519 |
| 116 | 04-Jan-22 | Omicron BA.1 | Yes | Possible Omicron (B.1.1.529) |  |  | Omicron (BA.1.1) | EPI_ISL_11512520 |
| 117 | 04-Jan-22 | Omicron BA.1 | Yes | Possible Omicron (B.1.1.529) | Omicron V2 panel | Omicron (BA.1) |  |  |
| 118 | 04-Jan-22 | Omicron BA.1 | Yes | Possible Omicron (B.1.1.529) |  |  | Omicron (BA.1) | EPI_ISL_11512521 |
| 119 | 04-Jan-22 | Omicron BA.1 | Yes | Possible Omicron (B.1.1.529) | Omicron V2 panel | Omicron (BA.1) |  |  |
| 120 | 04-Jan-22 | Omicron BA.1 | Yes | Possible Omicron (B.1.1.529) | Omicron V2 panel | Omicron (BA.1) |  |  |
| 121 | 04-Jan-22 | Omicron BA.1 | Yes | Possible Omicron (B.1.1.529) |  |  | Omicron (BA.1.1) | EPI_ISL_11563560 |
| 122 | 04-Jan-22 | Omicron BA.1 | Yes | Possible Omicron (B.1.1.529) |  |  | Omicron (BA.1) | EPI_ISL_11512522 |
| 123 | 04-Jan-22 | Omicron BA.1 | Yes | Possible Omicron (B.1.1.529) |  |  | Omicron (BA.1.1) | EPI_ISL_11512523 |
| 124 | 04-Jan-22 | Omicron BA.1 | Yes | Possible Omicron (B.1.1.529) |  |  | Omicron (BA.1.1) | EPI_ISL_11512524 |
| 125 | 04-Jan-22 | Omicron BA.1 | Yes | Possible Omicron (B.1.1.529) |  |  | Omicron (BA.1.1) | EPI_ISL_11512525 |
| 126 | 04-Jan-22 | Omicron BA.1 | Yes | Possible Omicron (B.1.1.529) |  |  | Omicron (BA.1.1) | EPI_ISL_11512526 |
| 127 | 04-Jan-22 | Omicron BA.1 | Yes | Possible Omicron (B.1.1.529) |  |  | Omicron (BA.1) | EPI_ISL_11512527 |
| 128 | 04-Jan-22 | Omicron BA.1 | Yes | Possible Omicron (B.1.1.529) |  |  | Omicron (BA.1) | EPI_ISL_11512528 |
| 129 | 04-Jan-22 | Omicron BA.1 | Yes | Possible Omicron (B.1.1.529) | Omicron V2 panel | Omicron (BA.1) |  |  |
| 130 | 05-Jan-22 | Omicron BA.1 | Yes | Possible Omicron (B.1.1.529) | Omicron V2 panel | Omicron (BA.1) |  |  |
| 131 | 05-Jan-22 | Delta | Yes | Non-omicron/ possible Delta | Omicron V2 panel | Delta (B.1.617.2) |  |  |
| 132 | 05-Jan-22 | Omicron BA.1 | Yes | Possible Omicron (B.1.1.529) | Omicron V2 panel | Omicron (BA.1) |  |  |
| 133 | 05-Jan-22 | Omicron BA.1 | Yes | Possible Omicron (B.1.1.529) | Omicron V2 panel | Omicron (BA.1) |  |  |
| 134 | 05-Jan-22 | Omicron BA.1 | Yes | Possible Omicron (B.1.1.529) | Omicron V2 panel | Omicron (BA.1) |  |  |
| 135 | 05-Jan-22 | Omicron BA.1 | Yes | Possible Omicron (B.1.1.529) | Omicron V2 panel | Omicron (BA.1) |  |  |
| 136 | 05-Jan-22 | Omicron BA.1 | Yes | Possible Omicron (B.1.1.529) | Omicron V2 panel | Omicron (BA.1) |  |  |
| 137 | 05-Jan-22 | Omicron BA.1 | Yes | Possible Omicron (B.1.1.529) | Omicron V2 panel | Omicron (BA.1) |  |  |
| 138 | 05-Jan-22 | Omicron BA.1 | Yes | Possible Omicron (B.1.1.529) | Omicron V2 panel | Omicron (BA.1) |  |  |
| 139 | 05-Jan-22 | Omicron BA.1 |  | Unidentified | Omicron V2 panel | Omicron (BA.1) |  |  |
| 140 | 05-Jan-22 | Delta | Yes | Non-omicron/ possible Delta | Omicron V2 panel | Delta (B.1.617.2) |  |  |
| 141 | 05-Jan-22 | Omicron BA.1 | Yes | Possible Omicron (B.1.1.529) | Omicron V2 panel | Omicron (BA.1) |  |  |
| 142 | 05-Jan-22 | Omicron BA.1 | Yes | Possible Omicron (B.1.1.529) | Omicron V2 panel | Omicron (BA.1) |  |  |
| 143 | 05-Jan-22 | Omicron BA.1 | Yes | Possible Omicron (B.1.1.529) | Omicron V2 panel | Omicron (BA.1) |  |  |
| 144 | 05-Jan-22 | Delta | Yes | Non-omicron/ possible Delta | Omicron V2 panel | Delta (B.1.617.2) |  |  |
| 145 | 05-Jan-22 | Omicron BA.1 | Yes | Possible Omicron (B.1.1.529) | Omicron V2 panel | Omicron (BA.1) |  |  |
| 146 | 05-Jan-22 | Omicron BA.1 | Yes | Possible Omicron (B.1.1.529) | Omicron V2 panel | Omicron (BA.1) |  |  |
| 147 | 05-Jan-22 | Omicron BA.1 | Yes | Possible Omicron (B.1.1.529) | Omicron V2 panel | Omicron (BA.1) |  |  |
| 148 | 05-Jan-22 | Omicron BA.1 |  | Unidentified | Omicron V2 panel | Omicron (BA.1) |  |  |
| 149 | 05-Jan-22 | Omicron BA.1 | Yes | Possible Omicron (B.1.1.529) | Omicron V2 panel | Omicron (BA.1) |  |  |
| 150 | 06-Jan-22 | Omicron BA.1 | Yes | Possible Omicron (B.1.1.529) | Omicron V2 panel | Omicron (BA.1) |  |  |
| 151 | 06-Jan-22 | Omicron BA.1 | Yes | Possible Omicron (B.1.1.529) | Omicron V2 panel | Omicron (BA.1) |  |  |
| 152 | 06-Jan-22 | Omicron BA.1 | Yes | Possible Omicron (B.1.1.529) | Omicron V2 panel | Omicron (BA.1) |  |  |
| 153 | 06-Jan-22 | Omicron BA.1 | Yes | Possible Omicron (B.1.1.529) | Omicron V2 panel | Omicron (BA.1) |  |  |
| 154 | 06-Jan-22 | Omicron BA.1 | Yes | Possible Omicron (B.1.1.529) | Omicron V2 panel | Omicron (BA.1) |  |  |
| 155 | 06-Jan-22 | Omicron BA.1 | Yes | Possible Omicron (B.1.1.529) | Omicron V2 panel | Omicron (BA.1) |  |  |
| 156 | 06-Jan-22 | Omicron BA.1 | Yes | Possible Omicron (B.1.1.529) | Omicron V2 panel | Omicron (BA.1) |  |  |
| 157 | 06-Jan-22 | Omicron BA.1 | Yes | Possible Omicron (B.1.1.529) | Omicron V2 panel | Omicron (BA.1) |  |  |
| 158 | 06-Jan-22 | Omicron BA.1 | Yes | Possible Omicron (B.1.1.529) | Omicron V2 panel | Omicron (BA.1) |  |  |
| 159 | 06-Jan-22 | Omicron BA.1 |  |  | Omicron V2 panel | Omicron (BA.1) |  |  |
| 160 | 06-Jan-22 | Omicron BA.1 | Yes | Possible Omicron (B.1.1.529) | Omicron V2 panel | Omicron (BA.1) |  |  |
| 161 | 06-Jan-22 | Omicron BA.1 | Yes | Possible Omicron (B.1.1.529) | Omicron V2 panel | Omicron (BA.1) |  |  |
| 162 | 06-Jan-22 | Omicron BA.1 | Yes | Possible Omicron (B.1.1.529) | Omicron V2 panel | Omicron (BA.1) |  |  |
| 163 | 06-Jan-22 | Omicron BA.1 |  | Possible Omicron (B.1.1.529) |  |  |  |  |
| 164 | 06-Jan-22 | Omicron BA.1 | Yes | Possible Omicron (B.1.1.529) | Omicron V2 panel | Omicron (BA.1) |  |  |
| 165 | 06-Jan-22 | Omicron BA.1 | Yes | Possible Omicron (B.1.1.529) | Omicron V2 panel | Omicron (BA.1) |  |  |
| 166 | 06-Jan-22 | Omicron BA.1 | Yes | Possible Omicron (B.1.1.529) | Omicron V2 panel | Omicron (BA.1) |  |  |
| 167 | 06-Jan-22 | Delta | Yes | Non-omicron/ possible Delta | Omicron V2 panel | Delta (B.1.617.2) |  |  |
| 168 | 06-Jan-22 | Omicron BA.1 | Yes | Possible Omicron (B.1.1.529) | Omicron V2 panel | Omicron (BA.1) |  |  |
| 169 | 06-Jan-22 | Omicron BA.1 | Yes | Possible Omicron (B.1.1.529) | Omicron V2 panel | Omicron (BA.1) |  |  |
| 170 | 07-Jan-22 | Omicron BA.1 | Yes | Possible Omicron (B.1.1.529) | Omicron V2 panel | Omicron (BA.1) |  |  |
| 171 | 07-Jan-22 | Omicron BA.1 | Yes | Possible Omicron (B.1.1.529) | Omicron V2 panel | Omicron (BA.1) |  |  |
| 172 | 07-Jan-22 | Omicron BA.1 | Yes | Possible Omicron (B.1.1.529) | Omicron V2 panel | Omicron (BA.1) |  |  |
| 173 | 07-Jan-22 | Omicron BA.1 | Yes | Possible Omicron (B.1.1.529) | Omicron V2 panel | Omicron (BA.1) |  |  |
| 174 | 10-Jan-22 | Omicron BA.1 | Yes | Possible Omicron (B.1.1.529) | Omicron V2 panel | Omicron (BA.1) |  |  |
| 175 | 10-Jan-22 | Omicron BA.1 | Yes | Possible Omicron (B.1.1.529) | Omicron V2 panel | Omicron (BA.1) |  |  |
| 176 | 10-Jan-22 | Omicron BA.1 |  | Possible Omicron (B.1.1.529) |  |  |  |  |
| 177 | 10-Jan-22 | Omicron BA.1 | Yes | Possible Omicron (B.1.1.529) | Omicron V2 panel | Omicron (BA.1) |  |  |
| 178 | 10-Jan-22 | Omicron BA.1 |  | Possible Omicron (B.1.1.529) |  |  |  |  |
| 179 | 10-Jan-22 | Omicron BA.1 | Yes | Possible Omicron (B.1.1.529) | Omicron V2 panel | Omicron (BA.1) |  |  |
| 180 | 10-Jan-22 | Omicron BA.1 | Yes | Possible Omicron (B.1.1.529) | Omicron V2 panel | Omicron (BA.1) |  |  |
| 181 | 10-Jan-22 | Delta | Yes | Non-omicron/ possible Delta | Omicron V2 panel | Delta (B.1.617.2) |  |  |
| 182 | 10-Jan-22 | Omicron BA.1 | Yes | Possible Omicron (B.1.1.529) | Omicron V2 panel | Omicron (BA.1) |  |  |
| 183 | 10-Jan-22 | Omicron BA.1 | Yes | Possible Omicron (B.1.1.529) | Omicron V2 panel | Omicron (BA.1) |  |  |
| 184 | 10-Jan-22 | Omicron BA.1 | Yes | Possible Omicron (B.1.1.529) | Omicron V2 panel | Omicron (BA.1) |  |  |
| 185 | 10-Jan-22 | Omicron BA.1 | Yes | Possible Omicron (B.1.1.529) | Omicron V2 panel | Omicron (BA.1) |  |  |
| 186 | 10-Jan-22 | Omicron BA.1 | Yes | Possible Omicron (B.1.1.529) | Omicron V2 panel | Omicron (BA.1) | Omicron (BA.1) | EPI_ISL_11327789 |
| 187 | 10-Jan-22 | Omicron BA.1 |  | Possible Omicron (B.1.1.529) |  |  |  |  |
| 188 | 10-Jan-22 | Omicron BA.1 |  |  | Omicron V2 panel | Omicron (BA.1) |  |  |
| 189 | 10-Jan-22 | Omicron BA.1 |  |  | Omicron V2 panel | Omicron (BA.1) |  |  |
| 190 | 10-Jan-22 | Delta |  |  | Omicron V2 panel | Delta (B.1.617.2) |  |  |
| 191 | 10-Jan-22 | Omicron BA.1 |  |  | Omicron V2 panel | Omicron (BA.1) |  |  |
| 192 | 10-Jan-22 | Omicron BA.1 |  |  | Omicron V2 panel | Omicron (BA.1) |  |  |
| 193 | 10-Jan-22 | Omicron BA.1 |  |  | Omicron V2 panel | Omicron (BA.1) |  |  |
| 194 | 10-Jan-22 | Omicron BA.1 |  |  | Omicron V2 panel | Omicron (BA.1) |  |  |
| 195 | 10-Jan-22 | Omicron BA.1 |  |  | Omicron V2 panel | Omicron (BA.1) |  |  |
| 196 | 10-Jan-22 | Omicron BA.1 |  |  | Omicron V2 panel | Omicron (BA.1) |  |  |
| 197 | 10-Jan-22 | Omicron BA.1 |  |  | Omicron V2 panel | Omicron (BA.1) |  |  |
| 198 | 10-Jan-22 | Omicron BA.1 |  |  | Omicron V2 panel | Omicron (BA.1) |  |  |
| 199 | 10-Jan-22 | Omicron BA.1 |  |  | Omicron V2 panel | Omicron (BA.1) |  |  |
| 200 | 10-Jan-22 | Omicron BA.1 |  |  | Omicron V2 panel | Omicron (BA.1) |  |  |
| 201 | 10-Jan-22 | Delta |  |  | Omicron V2 panel | Delta (B.1.617.2) |  |  |
| 202 | 10-Jan-22 | Omicron BA.1 |  |  | Omicron V2 panel | Omicron (BA.1) |  |  |
| 203 | 10-Jan-22 | Omicron BA.1 |  |  | Omicron V2 panel | Omicron (BA.1) |  |  |
| 204 | 10-Jan-22 | Omicron BA.1 |  |  | Omicron V2 panel | Omicron (BA.1) |  |  |
| 205 | 11-Jan-22 | Omicron BA.1 |  |  | Omicron V2 panel | Omicron (BA.1) |  |  |
| 206 | 11-Jan-22 | Omicron BA.1 |  |  | Omicron V2 panel | Omicron (BA.1) |  |  |
| 207 | 11-Jan-22 | Omicron BA.1 |  |  | Omicron V2 panel | Omicron (BA.1) |  |  |
| 208 | 11-Jan-22 | Omicron BA.1 |  |  | Omicron V2 panel | Omicron (BA.1) |  |  |
| 209 | 11-Jan-22 | Omicron BA.1 |  |  | Omicron V2 panel | Omicron (BA.1) |  |  |
| 210 | 11-Jan-22 | Omicron BA.1 |  |  | Omicron V2 panel | Omicron (BA.1) |  |  |
| 211 | 11-Jan-22 | Omicron BA.1 |  |  | Omicron V2 panel | Omicron (BA.1) |  |  |
| 212 | 11-Jan-22 | Omicron BA.1 |  |  | Omicron V2 panel | Omicron (BA.1) |  |  |
| 213 | 11-Jan-22 | Omicron BA.1 |  |  | Omicron V2 panel | Omicron (BA.1) |  |  |
| 214 | 11-Jan-22 | Omicron BA.1 |  |  | Omicron V2 panel | Omicron (BA.1) |  |  |
| 215 | 11-Jan-22 | Omicron BA.1 |  |  | Omicron V2 panel | Omicron (BA.1) |  |  |
| 216 | 11-Jan-22 | Omicron BA.1 |  |  | Omicron V2 panel | Omicron (BA.1) |  |  |
| 217 | 10-Jan-22 | Omicron BA.1 | Yes | Possible Omicron (B.1.1.529) | Omicron V2 panel | Omicron (BA.1) |  |  |
| 218 | 12-Jan-22 | Omicron BA.1 |  | Possible Omicron (B.1.1.529) |  |  |  |  |
| 219 | 12-Jan-22 | Omicron BA.1 |  |  | Omicron V2 panel | Omicron (BA.1) |  |  |
| 220 | 12-Jan-22 | Omicron BA.1 |  |  | Omicron V2 panel | Omicron (BA.1) |  |  |
| 221 | 12-Jan-22 | Omicron BA.1 |  |  | Omicron V2 panel | Omicron (BA.1) |  |  |
| 222 | 12-Jan-22 | Omicron BA.1 |  |  | Omicron V2 panel | Omicron (BA.1) |  |  |
| 223 | 12-Jan-22 | Omicron BA.1 |  |  | Omicron V2 panel | Omicron (BA.1) |  |  |
| 224 | 14-Jan-22 | Delta |  |  | Omicron V2 panel | Unidentified | Delta (AY.30) | EPI_ISL_17029347 |
| 225 | 14-Jan-22 | Omicron BA.1 |  |  | Omicron V2 panel | Omicron (BA.1) |  |  |
| 226 | 14-Jan-22 | Omicron BA.1 |  |  | Omicron V2 panel | Omicron (BA.1) |  |  |
| 227 | 14-Jan-22 | Omicron BA.1 |  |  | Omicron V2 panel | Omicron (BA.1) |  |  |
| 228 | 14-Jan-22 | Omicron BA.1 |  |  | Omicron V2 panel | Omicron (BA.1) |  |  |
| 229 | 14-Jan-22 | Omicron BA.1 |  |  | Omicron V2 panel | Omicron (BA.1) |  |  |
| 230 | 13-Jan-22 | Omicron BA.1 |  |  | Omicron V2 panel | Omicron (BA.1) |  |  |
| 231 | 13-Jan-22 | Omicron BA.1 |  | Possible Omicron (B.1.1.529) |  |  |  |  |
| 232 | 13-Jan-22 | Omicron BA.1 | Yes | Possible Omicron (B.1.1.529) | Omicron V2 panel | Omicron (BA.1) |  |  |
| 233 | 14-Jan-22 | Omicron BA.1 |  |  | Omicron V2 panel | Omicron (BA.1) |  |  |
| 234 | 14-Jan-22 | Omicron BA.1 |  |  | Omicron V2 panel | Omicron (BA.1) |  |  |
| 235 | 12-Jan-22 | Omicron BA.1 |  |  | Omicron V2 panel | Omicron (BA.1) |  |  |
| 236 | 12-Jan-22 | Omicron BA.1 |  |  | Omicron V2 panel | Omicron (BA.1) |  |  |
| 237 | 12-Jan-22 | Omicron BA.1 |  |  | Omicron V2 panel | Omicron (BA.1) |  |  |
| 238 | 10-Jan-22 | Omicron BA.1 |  |  | Omicron V2 panel | Omicron (BA.1) |  |  |
| 239 | 12-Jan-22 | Omicron BA.1 |  |  | Omicron V2 panel | Omicron (BA.1) |  |  |
| 240 | 07-Jan-22 | Omicron BA.1 |  |  | Omicron V2 panel | Omicron (BA.1) |  |  |
| 241 | 08-Jan-22 | Omicron BA.2 | Yes | Possible Omicron Stealth (BA.2) | Omicron V2 panel | Omicron (BA.2) | Omicron (BA.2) | EPI_ISL_9712645 |
| 242 | 08-Jan-22 | Omicron BA.2 | Yes | Possible Omicron Stealth (BA.2) | Omicron V2 panel | Omicron (BA.2) | Omicron (BA.2) | EPI_ISL_9712646 |
| 243 | 17-Jan-22 | Delta | Yes | Non-omicron/ possible Delta | Omicron V2 panel | Delta (B.1.617.2) | Delta (AY.112) | EPI_ISL_11327790 |
| 244 | 17-Jan-22 | Omicron BA.1 | No | Non-omicron/ possible Delta | Omicron V2 panel | Omicron (BA.1) |  |  |
| 245 | 17-Jan-22 | Omicron BA.1 |  |  | Omicron V2 panel | Omicron (BA.1) |  |  |
| 246 | 17-Jan-22 | Omicron BA.1 |  |  | Omicron V2 panel | Omicron (BA.1) |  |  |
| 247 | 17-Jan-22 | Omicron BA.1 |  |  | Omicron V2 panel | Omicron (BA.1) |  |  |
| 248 | 17-Jan-22 | Omicron BA.1 |  |  | Omicron V2 panel | Omicron (BA.1) |  |  |
| 249 | 17-Jan-22 | Omicron BA.1 |  |  | Omicron V2 panel | Omicron (BA.1) |  |  |
| 250 | 17-Jan-22 | Omicron BA.1 |  |  | Omicron V2 panel | Omicron (BA.1) |  |  |
| 251 | 17-Jan-22 | Omicron BA.1 |  |  | Omicron V2 panel | Omicron (BA.1) |  |  |
| 252 | 17-Jan-22 | Omicron BA.1 |  |  | Omicron V2 panel | Omicron (BA.1) |  |  |
| 253 | 17-Jan-22 | Omicron BA.1 |  |  | Omicron V2 panel | Omicron (BA.1) |  |  |
| 254 | 17-Jan-22 | Omicron BA.1 |  |  | Omicron V2 panel | Omicron (BA.1) |  |  |
| 255 | 17-Jan-22 | Omicron BA.1 |  |  | Omicron V2 panel | Omicron (BA.1) |  |  |
| 256 | 17-Jan-22 | Omicron BA.2 |  |  | Omicron V2 panel | Omicron (BA.2) |  |  |
| 257 | 17-Jan-22 | Omicron BA.1 |  |  | Omicron V2 panel | Omicron (BA.1) |  |  |
| 258 | 18-Jan-22 | Omicron BA.1 | Yes |  | Omicron V2 panel | Omicron (BA.1) | Omicron (BA.1) | EPI_ISL_11327791 |
| 259 | 18-Jan-22 | Delta | Yes | Non-omicron/ possible Delta | Omicron V2 panel | Delta (B.1.617.2) | Delta (AY.30) | EPI_ISL_17029348 |
| 260 | 18-Jan-22 | Omicron BA.1 | Yes | Possible Omicron (B.1.1.529) | Omicron V2 panel | Omicron (BA.1) |  |  |
| 261 | 18-Jan-22 | Omicron BA.1 |  |  | Omicron V2 panel | Omicron (BA.1) |  |  |
| 262 | 18-Jan-22 | Omicron BA.1 |  |  | Omicron V2 panel | Omicron (BA.1) |  |  |
| 263 | 18-Jan-22 | Omicron BA.1 |  |  | Omicron V2 panel | Omicron (BA.1) |  |  |
| 264 | 18-Jan-22 | Omicron BA.1 |  |  | Omicron V2 panel | Omicron (BA.1) |  |  |
| 265 | 18-Jan-22 | Omicron BA.1 | Yes | Possible Omicron (B.1.1.529) | Omicron V2 panel | Omicron (BA.1) | Failed |  |
| 266 | 19-Jan-22 | Omicron BA.1 |  |  | Omicron V2 panel | Omicron (BA.1) |  |  |
| 267 | 19-Jan-22 | Omicron BA.1 |  |  | Omicron V2 panel | Omicron (BA.1) |  |  |
| 268 | 19-Jan-22 | Omicron BA.1 |  |  | Omicron V2 panel | Omicron (BA.1) |  |  |
| 269 | 19-Jan-22 | Omicron BA.1 |  |  | Omicron V2 panel | Omicron (BA.1) |  |  |
| 270 | 19-Jan-22 | Omicron BA.1 |  |  | Omicron V2 panel | Omicron (BA.1) |  |  |
| 271 | 19-Jan-22 | Omicron BA.1 |  |  | Omicron V2 panel | Omicron (BA.1) |  |  |
| 272 | 20-Jan-22 | Omicron BA.1 |  |  | Omicron V2 panel | Omicron (BA.1) |  |  |
| 273 | 20-Jan-22 | Omicron BA.1 |  |  | Omicron V2 panel | Omicron (BA.1) |  |  |
| 274 | 20-Jan-22 | Omicron BA.1 | Yes |  | Omicron V2 panel | Omicron (BA.1) | Omicron (BA.1.1) | EPI_ISL_11327792 |
| 275 | 20-Jan-22 | Omicron BA.1 |  |  | Omicron V2 panel | Omicron (BA.1) |  |  |
| 276 | 20-Jan-22 | Omicron BA.1 |  |  | Omicron V2 panel | Omicron (BA.1) |  |  |
| 277 | 21-Jan-22 | Omicron BA.1 |  |  | Omicron V2 panel | Omicron (BA.1) |  |  |
| 278 | 21-Jan-22 | Omicron BA.1 |  |  | Omicron V2 panel | Omicron (BA.1) |  |  |
| 279 | 23-Jan-22 | Omicron BA.1 | Yes | Possible Omicron (B.1.1.529) | Omicron V2 panel | Omicron (BA.1) | Omicron (BA.1.1) | EPI_ISL_11327793 |
| 280 | 14-Jan-22 | Omicron BA.1 |  |  | Omicron V2 panel | Omicron (BA.1) |  |  |
| 281 | 17-Jan-22 | Omicron BA.1 |  |  | Omicron V2 panel | Omicron (BA.1) |  |  |
| 282 | 18-Jan-22 | Omicron BA.1 |  |  | Omicron V2 panel | Omicron (BA.1) |  |  |
| 283 | 18-Jan-22 | Omicron BA.1 |  |  | Omicron V2 panel | Omicron (BA.1) |  |  |
| 284 | 18-Jan-22 | Omicron BA.1 |  |  | Omicron V2 panel | Omicron (BA.1) |  |  |
| 285 | 18-Jan-22 | Omicron BA.1 |  |  | Omicron V2 panel | Omicron (BA.1) |  |  |
| 286 | 19-Jan-22 | Omicron BA.1 |  |  | Omicron V2 panel | Omicron (BA.1) |  |  |
| 287 | 19-Jan-22 | Omicron BA.1 |  |  | Omicron V2 panel | Omicron (BA.1) |  |  |
| 288 | 19-Jan-22 | Omicron BA.1 |  |  | Omicron V2 panel | Omicron (BA.1) |  |  |
| 289 | 19-Jan-22 | Omicron BA.1 |  |  | Omicron V2 panel | Omicron (BA.1) |  |  |
| 290 | 21-Jan-22 | Omicron BA.1 |  |  | Omicron V2 panel | Omicron (BA.1) |  |  |
| 291 | 15-Jan-22 | Omicron BA.1 |  |  | Omicron V2 panel | Omicron (BA.1) |  |  |
| 292 | 17-Jan-22 | Omicron BA.1 |  |  | Omicron V2 panel | Omicron (BA.1) |  |  |
| 293 | 22-Jan-22 | Omicron BA.1 |  |  | Omicron V2 panel | Omicron (BA.1) |  |  |
| 294 | 22-Jan-22 | Omicron BA.1 |  |  | Omicron V2 panel | Omicron (BA.1) |  |  |
| 295 | 22-Jan-22 | Omicron BA.1 |  |  | Omicron V2 panel | Omicron (BA.1) |  |  |
| 296 | 22-Jan-22 | Delta |  |  | Omicron V2 panel | Delta (B.1.617.2) |  |  |
| 297 | 22-Jan-22 | Omicron BA.1 |  |  | Omicron V2 panel | Omicron (BA.1) |  |  |
| 298 | 22-Jan-22 | Omicron BA.1 |  |  | Omicron V2 panel | Omicron (BA.1) |  |  |
| 299 | 23-Jan-22 | Omicron BA.1 |  |  | Omicron V2 panel | Omicron (BA.1) |  |  |
| 300 | 23-Jan-22 | Omicron BA.1 |  |  | Omicron V2 panel | Omicron (BA.1) |  |  |
| 301 | 23-Jan-22 | Omicron BA.1 |  |  | Omicron V2 panel | Omicron (BA.1) |  |  |
| 302 | 23-Jan-22 | Omicron BA.1 |  |  | Omicron V2 panel | Omicron (BA.1) |  |  |
| 303 | 24-Jan-22 | Omicron BA.1 |  |  | Omicron V2 panel | Omicron (BA.1) |  |  |
| 304 | 24-Jan-22 | Omicron BA.1 |  |  | Omicron V2 panel | Omicron (BA.1) |  |  |
| 305 | 25-Jan-22 | Omicron BA.1 |  |  | Omicron V2 panel | Omicron (BA.1) |  |  |
| 306 | 25-Jan-22 | Omicron BA.1 |  |  | Omicron V2 panel | Omicron (BA.1) |  |  |
| 307 | 27-Jan-22 | Omicron BA.1 | No | Non-omicron/ possible Delta | Omicron V2 panel | Omicron (BA.1) |  |  |
| 308 | 27-Jan-22 | Omicron BA.1 |  |  | Omicron V2 panel | Omicron (BA.1) |  |  |
| 309 | 27-Jan-22 | Omicron BA.1 |  |  | Omicron V2 panel | Omicron (BA.1) |  |  |
| 310 | 26-Jan-22 | Omicron BA.1 | Yes | Possible Omicron (B.1.1.529) | Omicron V2 panel | Omicron (BA.1) |  |  |
| 311 | 26-Jan-22 | Omicron BA.1 |  |  | Omicron V2 panel | Omicron (BA.1) |  |  |
| 312 | 26-Jan-22 | Omicron BA.1 |  |  | Omicron V2 panel | Omicron (BA.1) |  |  |
| 313 | 26-Jan-22 | Omicron BA.1 |  |  | Omicron V2 panel | Omicron (BA.1) |  |  |
| 314 | 26-Jan-22 | Omicron BA.1 | Yes | Possible Omicron (B.1.1.529) | Omicron V2 panel | Omicron (BA.1) |  |  |
| 315 | 26-Jan-22 | Omicron BA.1 | Yes | Possible Omicron (B.1.1.529) | Omicron V2 panel | Omicron (BA.1) |  |  |
| 316 | 26-Jan-22 | Omicron BA.1 |  |  | Omicron V2 panel | Omicron (BA.1) |  |  |
| 317 | 26-Jan-22 | Omicron BA.1 |  |  | Omicron V2 panel | Omicron (BA.1) |  |  |
| 318 | 28-Jan-22 | Omicron BA.1 | Yes | Possible Omicron (B.1.1.529) | Omicron V2 panel | Omicron (BA.1) | Omicron (BA.1) | EPI_ISL_11327794 |
| 319 | 30-Jan-22 | Omicron BA.1 | Yes | Possible Omicron (B.1.1.529) | Omicron V2 panel | Omicron (BA.1) |  |  |
| 320 | 27-Jan-22 | Omicron BA.1 | Yes | Possible Omicron (B.1.1.529) | Omicron V2 panel | Omicron (BA.1) |  |  |
| 321 | 30-Jan-22 | Omicron BA.1 | Yes | Possible Omicron (B.1.1.529) | Omicron V2 panel | Omicron (BA.1) |  |  |
| 322 | 31-Jan-22 | Omicron BA.1 |  |  | Omicron V2 panel | Omicron (BA.1) |  |  |
| 323 | 31-Jan-22 | Omicron BA.1 |  |  | Omicron V2 panel | Omicron (BA.1) |  |  |
| 324 | 31-Jan-22 | Omicron BA.1 |  |  | Omicron V2 panel | Omicron (BA.1) |  |  |
| 325 | 02-Feb-22 | Omicron BA.1 | Yes | Possible Omicron (B.1.1.529) | Omicron V2 panel | Omicron (BA.1) |  |  |
| 326 | 02-Feb-22 | Omicron BA.1 |  |  | Omicron V2 panel | Omicron (BA.1) |  |  |
| 327 | 02-Feb-22 | Omicron BA.2 | Yes |  | Omicron V2 panel | Omicron (BA.2) | Omicron (BA.2) | EPI_ISL_11563137 |
| 328 | 02-Feb-22 | Omicron BA.1 | Yes | Possible Omicron (B.1.1.529) | Omicron V2 panel | Omicron (BA.1) | Omicron (BA.1.1) | EPI_ISL_11327795 |
| 329 | 02-Feb-22 | Omicron BA.1 |  |  | Omicron V2 panel | Omicron (BA.1) |  |  |
| 330 | 02-Feb-22 | Omicron BA.2 | Yes |  | Omicron V2 panel | Omicron (BA.2) | Omicron (BA.2) | EPI_ISL_11563138 |
| 331 | 02-Feb-22 | Omicron BA.1 |  |  | Omicron V2 panel | Omicron (BA.1) |  |  |
| 332 | 02-Feb-22 | Omicron BA.1 |  |  | Omicron V2 panel | Omicron (BA.1) |  |  |
| 333 | 02-Feb-22 | Omicron BA.1 |  |  | Omicron V2 panel | Omicron (BA.1) |  |  |
| 334 | 02-Feb-22 | Omicron BA.1 |  |  | Omicron V2 panel | Omicron (BA.1) |  |  |
| 335 | 03-Feb-22 | Omicron BA.1 |  |  | Omicron V2 panel | Omicron (BA.1) |  |  |
| 336 | 03-Feb-22 | Omicron BA.1 |  |  | Omicron V2 panel | Omicron (BA.1) |  |  |
| 337 | 03-Feb-22 | Omicron BA.1 |  |  | Omicron V2 panel | Omicron (BA.1) |  |  |
| 338 | 03-Feb-22 | Omicron BA.1 | Yes |  | Omicron V2 panel | Omicron (BA.1) | Omicron (BA.1.1) | EPI_ISL_11327796 |
| 339 | 03-Feb-22 | Omicron BA.2 | Yes |  | Omicron V2 panel | Omicron (BA.2) | Omicron (BA.2) | EPI_ISL_11563139 |
| 340 | 03-Feb-22 | Omicron BA.1 |  |  | Omicron V2 panel | Omicron (BA.1) |  |  |
| 341 | 03-Feb-22 | Omicron BA.2 | Yes |  | Omicron V2 panel | Omicron (BA.2) | Omicron (BA.2) | EPI_ISL_11563140 |
| 342 | 03-Feb-22 | Omicron BA.2 | Yes |  | Omicron V2 panel | Omicron (BA.2) | Omicron (BA.2) | EPI_ISL_11563141 |
| 343 | 03-Feb-22 | Omicron BA.1 | Yes | Possible Omicron (B.1.1.529) | Omicron V2 panel | Omicron (BA.1) |  |  |
| 344 | 24-Jan-22 | Omicron BA.1 |  |  | Omicron V2 panel | Omicron (BA.1) |  |  |
| 345 | 26-Jan-22 | Omicron BA.1 |  |  | Omicron V2 panel | Omicron (BA.1) |  |  |
| 346 | 27-Jan-22 | Omicron BA.1 |  |  | Omicron V2 panel | Omicron (BA.1) |  |  |
| 347 | 29-Jan-22 | Omicron BA.1 |  |  | Omicron V2 panel | Omicron (BA.1) |  |  |
| 348 | 29-Jan-22 | Omicron BA.1 |  |  | Omicron V2 panel | Omicron (BA.1) |  |  |
| 349 | 30-Jan-22 | Omicron BA.1 |  |  | Omicron V2 panel | Omicron (BA.1) |  |  |
| 350 | 05-Feb-22 | Omicron BA.1 |  |  | Omicron V2 panel | Omicron (BA.1) |  |  |
| 351 | 05-Feb-22 | Omicron BA.1 |  |  | Omicron V2 panel | Omicron (BA.1) |  |  |
| 352 | 05-Feb-22 | Omicron BA.1 |  |  | Omicron V2 panel | Omicron (BA.1) |  |  |
| 353 | 05-Feb-22 | Omicron BA.1 |  |  | Omicron V2 panel | Omicron (BA.1) |  |  |
| 354 | 05-Feb-22 | Omicron BA.1 |  |  | Omicron V2 panel | Omicron (BA.1) |  |  |
| 355 | 05-Feb-22 | Omicron BA.1 |  |  | Omicron V2 panel | Omicron (BA.1) |  |  |
| 356 | 05-Feb-22 | Omicron BA.1 |  |  | Omicron V2 panel | Omicron (BA.1) |  |  |
| 357 | 05-Feb-22 | Omicron BA.1 |  |  | Omicron V2 panel | Omicron (BA.1) |  |  |
| 358 | 05-Feb-22 | Omicron BA.1 |  |  | Omicron V2 panel | Omicron (BA.1) |  |  |
| 359 | 05-Feb-22 | Omicron BA.1 |  |  | Omicron V2 panel | Omicron (BA.1) |  |  |
| 360 | 05-Feb-22 | Omicron BA.1 |  |  | Omicron V2 panel | Omicron (BA.1) |  |  |
| 361 | 07-Feb-22 | Omicron BA.1 | Yes |  | Omicron V2 panel | Omicron (BA.1) | Omicron (BA.1) | EPI_ISL_11327797 |
| 362 | 07-Feb-22 | Omicron BA.1 |  |  | Omicron V2 panel | Omicron (BA.1) |  |  |
| 363 | 07-Feb-22 | Omicron BA.1 |  |  | Omicron V2 panel | Omicron (BA.1) | Failed |  |
| 364 | 06-Feb-22 | Omicron BA.1 |  |  | Omicron V2 panel | Omicron (BA.1) |  |  |
| 365 | 06-Feb-22 | Omicron BA.1 |  |  | Omicron V2 panel | Omicron (BA.1) |  |  |
| 366 | 06-Feb-22 | Omicron BA.1 |  |  | Omicron V2 panel | Omicron (BA.1) |  |  |
| 367 | 06-Feb-22 | Omicron BA.1 |  |  | Omicron V2 panel | Omicron (BA.1) |  |  |
| 368 | 06-Feb-22 | Omicron BA.1 |  |  | Omicron V2 panel | Omicron (BA.1) |  |  |
| 369 | 06-Feb-22 | Omicron BA.1 |  |  | Omicron V2 panel | Omicron (BA.1) |  |  |
| 370 | 06-Feb-22 | Omicron BA.1 |  |  | Omicron V2 panel | Omicron (BA.1) |  |  |
| 371 | 06-Feb-22 | Omicron BA.2 |  |  | Omicron V2 panel | Omicron (BA.2) |  |  |
| 372 | 07-Feb-22 | Omicron BA.1 |  |  | Omicron V2 panel | Omicron (BA.1) |  |  |
| 373 | 07-Feb-22 | Omicron BA.1 | Yes |  | Omicron V2 panel | Omicron (BA.1) | Omicron (BA.1) | EPI_ISL_11327798 |
| 374 | 07-Feb-22 | Omicron BA.1 | Yes |  | Omicron V2 panel | Omicron (BA.1) | Omicron (BA.1.1) | EPI_ISL_11327799 |
| 375 | 07-Feb-22 | Omicron BA.1 |  |  | Omicron V2 panel | Omicron (BA.1) |  |  |
| 376 | 07-Feb-22 | Omicron BA.1 |  |  | Omicron V2 panel | Omicron (BA.1) |  |  |
| 377 | 07-Feb-22 | Omicron BA.1 |  |  | Omicron V2 panel | Omicron (BA.1) |  |  |
| 378 | 07-Feb-22 | Omicron BA.1 |  |  | Omicron V2 panel | Omicron (BA.1) |  |  |
| 379 | 07-Feb-22 | Omicron BA.2 |  |  | Omicron V2 panel | Omicron (BA.2) |  |  |
| 380 | 08-Feb-22 | Omicron BA.1 |  |  | Omicron V2 panel | Omicron (BA.1) |  |  |
| 381 | 08-Feb-22 | Omicron BA.1 |  |  | Omicron V2 panel | Omicron (BA.1) |  |  |
| 382 | 08-Feb-22 | Omicron BA.1 |  |  | Omicron V2 panel | Omicron (BA.1) |  |  |
| 383 | 08-Feb-22 | Omicron BA.1 | Yes |  | Omicron V2 panel | Omicron (BA.1) | Omicron (BA.1) | EPI_ISL_11327800 |
| 384 | 08-Feb-22 | Omicron BA.1 | Yes |  | Omicron V2 panel | Omicron (BA.1) | Omicron (BA.1.1) | EPI_ISL_11327801 |
| 385 | 08-Feb-22 | Omicron BA.2 |  |  | Omicron V2 panel | Omicron (BA.2) |  |  |
| 386 | 09-Feb-22 | Omicron BA.1 |  |  | Omicron V2 panel | Omicron (BA.1) |  |  |
| 387 | 24-Jan-22 | Omicron BA.1 | Yes |  | Omicron V2 panel | Omicron (BA.1) | Omicron (BA.1.1) | EPI_ISL_11327802 |
| 388 | 10-Feb-22 | Omicron BA.1 |  |  | Omicron V2 panel | Omicron (BA.1) | Failed |  |
| 389 | 09-Feb-22 | Omicron BA.1 |  |  | Omicron V2 panel | Omicron (BA.1) |  |  |
| 390 | 09-Feb-22 | Omicron BA.2 | Yes | Possible Omicron Stealth (BA.2) | Omicron V2 panel | Omicron (BA.2) |  |  |
| 391 | 09-Feb-22 | Omicron BA.1 |  |  | Omicron V2 panel | Omicron (BA.1) |  |  |
| 392 | 09-Feb-22 | Omicron BA.2 | Yes |  | Omicron V2 panel | Omicron (BA.2) | Omicron (BA.2) | EPI_ISL_11609742 |
| 393 | 09-Feb-22 | Omicron BA.1 |  |  | Omicron V2 panel | Omicron (BA.1) |  |  |
| 394 | 09-Feb-22 | Omicron BA.1 |  |  | Omicron V2 panel | Omicron (BA.1) |  |  |
| 395 | 09-Feb-22 | Omicron BA.1 |  |  | Omicron V2 panel | Omicron (BA.1) |  |  |
| 396 | 09-Feb-22 | Omicron BA.2 |  |  | Omicron V2 panel | Omicron (BA.2) |  |  |
| 397 | 09-Feb-22 | Omicron BA.1 |  |  | Omicron V2 panel | Omicron (BA.1) |  |  |
| 398 | 09-Feb-22 | Omicron BA.1 |  |  | Omicron V2 panel | Omicron (BA.1) |  |  |
| 399 | 09-Feb-22 | Omicron BA.1 |  |  | Omicron V2 panel | Omicron (BA.1) |  |  |
| 400 | 09-Feb-22 | Omicron BA.2 | Yes |  | Omicron V2 panel | Omicron (BA.2) | Omicron (BA.2) | EPI_ISL_11609743 |
| 401 | 09-Feb-22 | Omicron BA.2 | Yes |  | Omicron V2 panel | Omicron (BA.2) | Omicron (BA.2) | EPI_ISL_11609744 |
| 402 | 09-Feb-22 | Omicron BA.1 |  |  | Omicron V2 panel | Omicron (BA.1) |  |  |
| 403 | 09-Feb-22 | Omicron BA.1 |  |  | Omicron V2 panel | Omicron (BA.1) |  |  |
| 404 | 09-Feb-22 | Omicron BA.1 |  |  | Omicron V2 panel | Omicron (BA.1) |  |  |
| 405 | 09-Feb-22 | Omicron BA.1 |  |  | Omicron V2 panel | Omicron (BA.1) |  |  |
| 406 | 09-Feb-22 | Omicron BA.2 |  |  | Omicron V2 panel | Omicron (BA.2) |  |  |
| 407 | 09-Feb-22 | Omicron BA.1 |  |  | Omicron V2 panel | Omicron (BA.1) |  |  |
| 408 | 09-Feb-22 | Omicron BA.1 |  |  | Omicron V2 panel | Omicron (BA.1) |  |  |
| 409 | 09-Feb-22 | Omicron BA.1 |  |  | Omicron V2 panel | Omicron (BA.1) |  |  |
| 410 | 09-Feb-22 | Omicron BA.1 |  |  | Omicron V2 panel | Omicron (BA.1) |  |  |
| 411 | 09-Feb-22 | Omicron BA.1 | Yes |  | Omicron V2 panel | Omicron (BA.1) | Omicron (BA.1.1) | EPI_ISL_11327803 |
| 412 | 14-Feb-22 | Omicron BA.1 | Yes |  | Omicron V2 panel | Omicron (BA.1) | Omicron (BA.1.1) | EPI_ISL_11327804 |
| 413 | 14-Feb-22 | Omicron BA.1 |  |  | Omicron V2 panel | Omicron (BA.1) |  |  |
| 414 | 14-Feb-22 | Omicron BA.1 |  |  | Omicron V2 panel | Omicron (BA.1) |  |  |
| 415 | 14-Feb-22 | Omicron BA.1 |  |  | Omicron V2 panel | Omicron (BA.1) |  |  |
| 416 | 14-Feb-22 | Omicron BA.1 |  |  | Omicron V2 panel | Omicron (BA.1) |  |  |
| 417 | 14-Feb-22 | Omicron BA.1 |  |  | Omicron V2 panel | Omicron (BA.1) |  |  |
| 418 | 14-Feb-22 | Omicron BA.1 |  |  | Omicron V2 panel | Omicron (BA.1) |  |  |
| 419 | 14-Feb-22 | Omicron BA.1 |  |  | Omicron V2 panel | Omicron (BA.1) |  |  |
| 420 | 14-Feb-22 | Omicron BA.2 | Yes |  | Omicron V2 panel | Omicron (BA.2) | Omicron (BA.2) | EPI_ISL_11609767 |
| 421 | 14-Feb-22 | Omicron BA.2 | Yes |  | Omicron V2 panel | Omicron (BA.2) | Omicron (BA.2) | EPI_ISL_11609745 |
| 422 | 14-Feb-22 | Omicron BA.1 |  |  | Omicron V2 panel | Omicron (BA.1) |  |  |
| 423 | 15-Feb-22 | Omicron BA.2 |  |  | Omicron V2 panel | Omicron (BA.2) |  |  |
| 424 | 15-Feb-22 | Omicron BA.1 |  |  | Omicron V2 panel | Omicron (BA.1) |  |  |
| 425 | 15-Feb-22 | Omicron BA.2 | Yes |  | Omicron V2 panel | Omicron (BA.2) | Omicron (BA.2) | EPI_ISL_11609770 |
| 426 | 15-Feb-22 | Omicron BA.1 |  |  | Omicron V2 panel | Omicron (BA.1) |  |  |
| 427 | 17-Feb-22 | Omicron BA.1 |  |  | Omicron V2 panel | Omicron (BA.1) |  |  |
| 428 | 17-Feb-22 | Omicron BA.1 |  |  | Omicron V2 panel | Omicron (BA.1) |  |  |
| 429 | 17-Feb-22 | Omicron BA.1 |  |  | Omicron V2 panel | Omicron (BA.1) |  |  |
| 430 | 17-Feb-22 | Omicron BA.1 |  |  | Omicron V2 panel | Omicron (BA.1) |  |  |
| 431 | 17-Feb-22 | Omicron BA.1 |  |  | Omicron V2 panel | Omicron (BA.1) |  |  |
| 432 | 17-Feb-22 | Omicron BA.2 | Yes | Possible Omicron Stealth (BA.2) | Omicron V2 panel | Omicron (BA.2) |  |  |
| 433 | 17-Feb-22 | Omicron BA.1 |  |  | Omicron V2 panel | Omicron (BA.1) |  |  |
| 434 | 20-Feb-22 | Omicron BA.1 | Yes | Possible Omicron (B.1.1.529) | Omicron V2 panel | Omicron (BA.1) |  |  |
| 435 | 20-Feb-22 | Omicron BA.2 | Yes | Possible Omicron Stealth (BA.2) | Omicron V2 panel | Omicron (BA.2) | Omicron (BA.2) | EPI_ISL_11609764 |
| 436 | 20-Feb-22 | Omicron BA.1 | Yes | Possible Omicron (B.1.1.529) | Omicron V2 panel | Omicron (BA.1) |  |  |
| 437 | 20-Feb-22 | Omicron BA.1 | No | Possible Omicron Stealth (BA.2) | Omicron V2 panel | Omicron (BA.1) |  |  |
| 438 | 20-Feb-22 | Omicron BA.2 | Yes | Possible Omicron Stealth (BA.2) | Omicron V2 panel | Omicron (BA.2.2) | Omicron (BA.2) | EPI_ISL_11609765 |
| 439 | 20-Feb-22 | Omicron BA.2 | Yes | Possible Omicron Stealth (BA.2) | Omicron V2 panel | Omicron (BA.2) | Omicron (BA.2) | EPI_ISL_11609746 |
| 440 | 20-Feb-22 | Omicron BA.1 | No | Possible Omicron Stealth (BA.2) | Omicron V2 panel | Omicron (BA.1) |  |  |
| 441 | 20-Feb-22 | Omicron BA.1 | Yes | Possible Omicron (B.1.1.529) | Omicron V2 panel | Omicron (BA.1) |  |  |
| 442 | 20-Feb-22 | Omicron BA.1 | Yes | Possible Omicron (B.1.1.529) | Omicron V2 panel | Omicron (BA.1) |  |  |
| 443 | 20-Feb-22 | Omicron BA.1 | Yes | Possible Omicron (B.1.1.529) | Omicron V2 panel | Omicron (BA.1) |  |  |
| 444 | 20-Feb-22 | Omicron BA.1 | Yes | Possible Omicron (B.1.1.529) | Omicron V2 panel | Omicron (BA.1) |  |  |
| 445 | 20-Feb-22 | Omicron BA.2 | Yes | Possible Omicron Stealth (BA.2) | Omicron V2 panel | Omicron (BA.2) | Omicron (BA.2) | EPI_ISL_11609747 |
| 446 | 20-Feb-22 | Omicron BA.2 | Yes | Possible Omicron Stealth (BA.2) | Omicron V2 panel | Omicron (BA.2) |  |  |
| 447 | 20-Feb-22 | Omicron BA.2 | Yes | Possible Omicron Stealth (BA.2) | Omicron V2 panel | Omicron (BA.2) |  |  |
| 448 | 17-Feb-22 | Omicron BA.1 | Yes | Possible Omicron (B.1.1.529) | Omicron V2 panel | Omicron (BA.1) | Omicron (BA.1) | EPI_ISL_11327805 |
| 449 | 13-Feb-22 | Omicron BA.1 | Yes | Possible Omicron (B.1.1.529) | Omicron V2 panel | Omicron (BA.1) | Omicron (BA.1.1) | EPI_ISL_11327806 |
| 450 | 17-Feb-22 | Omicron BA.1 | Yes | Possible Omicron (B.1.1.529) | Omicron V2 panel | Omicron (BA.1) |  |  |
| 451 | 21-Feb-22 | Omicron BA.2 | Yes | Possible Omicron Stealth (BA.2) | Omicron V2 panel | Omicron (BA.2) |  |  |
| 452 | 23-Feb-22 | Omicron BA.2 | Yes | Possible Omicron Stealth (BA.2) | Omicron V2 panel | Omicron (BA.2) |  |  |
| 453 | 23-Feb-22 | Omicron BA.1 | Yes | Possible Omicron (B.1.1.529) | Omicron V2 panel | Omicron (BA.1) |  |  |
| 454 | 23-Feb-22 | Omicron BA.2 | Yes | Possible Omicron Stealth (BA.2) | Omicron V2 panel | Omicron (BA.2) | Omicron (BA.2) | EPI_ISL_11609768 |
| 455 | 23-Feb-22 | Omicron BA.2 | Yes | Possible Omicron Stealth (BA.2) | Omicron V2 panel | Omicron (BA.2) |  |  |
| 456 | 23-Feb-22 | Omicron BA.1 | Yes | Possible Omicron (B.1.1.529) | Omicron V2 panel | Omicron (BA.1) |  |  |
| 457 | 23-Feb-22 | Omicron BA.1 |  | Unidentified | Omicron V2 panel | Omicron (BA.1) |  |  |
| 458 | 23-Feb-22 | Omicron BA.1 | Yes | Possible Omicron (B.1.1.529) | Omicron V2 panel | Omicron (BA.1) |  |  |
| 459 | 23-Feb-22 | Omicron BA.2 | Yes | Possible Omicron Stealth (BA.2) | Omicron V2 panel | Omicron (BA.2) | Omicron (BA.2) | EPI_ISL_11609748 |
| 460 | 23-Feb-22 | Omicron BA.1 | Yes | Possible Omicron (B.1.1.529) | Omicron V2 panel | Omicron (BA.1) |  |  |
| 461 | 23-Feb-22 | Omicron BA.2 | Yes | Possible Omicron Stealth (BA.2) | Omicron V2 panel | Omicron (BA.2) |  |  |
| 462 | 23-Feb-22 | Omicron BA.1 | Yes | Possible Omicron (B.1.1.529) | Omicron V2 panel | Omicron (BA.1) |  |  |
| 463 | 23-Feb-22 | Omicron BA.1 | Yes | Possible Omicron (B.1.1.529) | Omicron V2 panel | Omicron (BA.1) |  |  |
| 464 | 23-Feb-22 | Omicron BA.2 | Yes | Possible Omicron Stealth (BA.2) | Omicron V2 panel | Omicron (BA.2) |  |  |
| 465 | 23-Feb-22 | Omicron BA.1 | Yes | Possible Omicron (B.1.1.529) | Omicron V2 panel | Omicron (BA.1) |  |  |
| 466 | 23-Feb-22 | Omicron BA.1 | Yes | Possible Omicron (B.1.1.529) | Omicron V2 panel | Omicron (BA.1) |  |  |
| 467 | 24-Feb-22 | Omicron BA.1 | Yes | Possible Omicron (B.1.1.529) | Omicron V2 panel | Omicron (BA.1) | Omicron (BA.1.1) | EPI_ISL_11327807 |
| 468 | 24-Feb-22 | Omicron BA.1 | Yes | Possible Omicron (B.1.1.529) | Omicron V2 panel | Omicron (BA.1) | Omicron (BA.1.1) | EPI_ISL_11609761 |
| 469 | 24-Feb-22 | Omicron BA.1 | Yes | Possible Omicron (B.1.1.529) | Omicron V2 panel | Omicron (BA.1) | Omicron (BA.1) | EPI_ISL_11327808 |
| 470 | 24-Feb-22 | Omicron BA.2 | Yes | Possible Omicron Stealth (BA.2) | Omicron V2 panel | Omicron (BA.2) | Omicron (BA.2) | EPI_ISL_11609749 |
| 471 | 24-Feb-22 | Omicron BA.1 | Yes | Possible Omicron (B.1.1.529) | Omicron V2 panel | Omicron (BA.1) |  |  |
| 472 | 24-Feb-22 | Omicron BA.1 | No | Possible Omicron Stealth (BA.2) | Omicron V2 panel | Omicron (BA.1) |  |  |
| 473 | 24-Feb-22 | Omicron BA.2 | Yes | Possible Omicron Stealth (BA.2) | Omicron V2 panel | Omicron (BA.2) |  |  |
| 474 | 24-Feb-22 | Omicron BA.1 | Yes | Possible Omicron (B.1.1.529) | Omicron V2 panel | Omicron (BA.1) |  |  |
| 475 | 24-Feb-22 | Omicron BA.2 | Yes | Possible Omicron Stealth (BA.2) | Omicron V2 panel | Omicron (BA.2) |  |  |
| 476 | 24-Feb-22 | Omicron BA.2 | Yes | Possible Omicron Stealth (BA.2) | Omicron V2 panel | Omicron (BA.2) |  |  |
| 477 | 24-Feb-22 | Omicron BA.1 | Yes | Possible Omicron (B.1.1.529) | Omicron V2 panel | Omicron (BA.1) |  |  |
| 478 | 24-Feb-22 | Omicron BA.2 | Yes | Possible Omicron Stealth (BA.2) | Omicron V2 panel | Omicron (BA.2) |  |  |
| 479 | 24-Feb-22 | Omicron BA.2 | Yes | Possible Omicron Stealth (BA.2) | Omicron V2 panel | Omicron (BA.2) |  |  |
| 480 | 24-Feb-22 | Omicron BA.1 | Yes | Possible Omicron (B.1.1.529) | Omicron V2 panel | Omicron (BA.1) |  |  |
| 481 | 25-Feb-22 | Omicron BA.1 | Yes | Possible Omicron (B.1.1.529) | Omicron V2 panel | Omicron (BA.1) | Failed |  |
| 482 | 25-Feb-22 | Omicron BA.2 | Yes | Possible Omicron Stealth (BA.2) | Omicron V2 panel | Omicron (BA.2) | Omicron (BA.2) | EPI_ISL_11609750 |
| 483 | 28-Feb-22 | Omicron BA.2 | Yes | Possible Omicron Stealth (BA.2) | Omicron V2 panel | Omicron (BA.2) |  |  |
| 484 | 28-Feb-22 | Omicron BA.2 | Yes | Possible Omicron Stealth (BA.2) | Omicron V2 panel | Omicron (BA.2) |  |  |
| 485 | 28-Feb-22 | Omicron BA.2 | Yes | Possible Omicron Stealth (BA.2) | Omicron V2 panel | Omicron (BA.2) |  |  |
| 486 | 28-Feb-22 | Omicron BA.2 | Yes | Possible Omicron Stealth (BA.2) | Omicron V2 panel | Omicron (BA.2) |  |  |
| 487 | 28-Feb-22 | Omicron BA.1 | Yes | Possible Omicron (B.1.1.529) | Omicron V2 panel | Omicron (BA.1) |  |  |
| 488 | 28-Feb-22 | Omicron BA.2 | Yes | Possible Omicron Stealth (BA.2) | Omicron V2 panel | Omicron (BA.2) | Omicron (BA.2) | EPI_ISL_11327809 |
| 489 | 28-Feb-22 | Omicron BA.2 | Yes | Possible Omicron Stealth (BA.2) | Omicron V2 panel | Omicron (BA.2) |  |  |
| 490 | 28-Feb-22 | Omicron BA.2 | Yes | Possible Omicron Stealth (BA.2) | Omicron V2 panel | Omicron (BA.2) |  |  |
| 491 | 28-Feb-22 | Omicron BA.2 | Yes | Possible Omicron Stealth (BA.2) | Omicron V2 panel | Omicron (BA.2) | Failed |  |
| 492 | 28-Feb-22 | Omicron BA.2 | Yes | Possible Omicron Stealth (BA.2) | Omicron V2 panel | Omicron (BA.2) | Omicron (BA.2) | EPI_ISL_11609751 |
| 493 | 01-Mar-22 | Omicron BA.1 |  | Possible Omicron (B.1.1.529) |  |  |  |  |
| 494 | 01-Mar-22 | Omicron BA.1 |  | Possible Omicron (B.1.1.529) |  |  |  |  |
| 495 | 01-Mar-22 | Omicron BA.1 |  | Possible Omicron (B.1.1.529) |  |  |  |  |
| 496 | 01-Mar-22 | Omicron BA.2 |  | Possible Omicron Stealth (BA.2) |  |  |  |  |
| 497 | 01-Mar-22 | Omicron BA.1 |  | Possible Omicron (B.1.1.529) |  |  |  |  |
| 498 | 01-Mar-22 | Omicron BA.2 |  | Possible Omicron Stealth (BA.2) |  |  |  |  |
| 499 | 01-Mar-22 | Omicron BA.2 |  | Possible Omicron Stealth (BA.2) |  |  |  |  |
| 500 | 01-Mar-22 | Omicron BA.2 |  | Possible Omicron Stealth (BA.2) |  |  |  |  |
| 501 | 01-Mar-22 | Omicron BA.2 |  | Possible Omicron Stealth (BA.2) |  |  |  |  |
| 502 | 02-Mar-22 | Omicron BA.2 | Yes | Possible Omicron Stealth (BA.2) |  |  | Omicron (BA.2) | EPI_ISL_11609766 |
| 503 | 03-Feb-22 | Omicron BA.1 | Yes | Possible Omicron (B.1.1.529) |  |  | Omicron (BA.1.1) | EPI_ISL_11327810 |
| 504 | 02-Mar-22 | Omicron BA.1 |  | Possible Omicron (B.1.1.529) |  |  |  |  |
| 505 | 02-Mar-22 | Omicron BA.1 |  | Possible Omicron (B.1.1.529) |  |  |  |  |
| 506 | 02-Mar-22 | Omicron BA.2 |  | Possible Omicron Stealth (BA.2) |  |  |  |  |
| 507 | 02-Mar-22 | Omicron BA.1 |  | Possible Omicron (B.1.1.529) |  |  |  |  |
| 508 | 02-Mar-22 | Omicron BA.2 |  | Possible Omicron Stealth (BA.2) |  |  |  |  |
| 509 | 02-Mar-22 | Omicron BA.2 |  | Possible Omicron Stealth (BA.2) |  |  |  |  |
| 510 | 02-Mar-22 | Omicron BA.2 |  | Possible Omicron Stealth (BA.2) |  |  |  |  |
| 511 | 02-Mar-22 | Delta |  | Non-omicron/ possible Delta |  |  |  |  |
| 512 | 02-Mar-22 | Omicron BA.2 |  | Possible Omicron Stealth (BA.2) |  |  |  |  |
| 513 | 02-Mar-22 | Omicron BA.1 |  | Possible Omicron (B.1.1.529) |  |  |  |  |
| 514 | 03-Mar-22 | Omicron BA.2 |  | Possible Omicron Stealth (BA.2) |  |  |  |  |
| 515 | 03-Mar-22 | Omicron BA.1 |  | Possible Omicron (B.1.1.529) |  |  |  |  |
| 516 | 04-Mar-22 | Omicron BA.2 | Yes | Possible Omicron Stealth (BA.2) |  |  | Omicron (BA.2) | EPI_ISL_11609763 |
| 517 | 04-Mar-22 | Omicron BA.2 |  | Possible Omicron Stealth (BA.2) |  |  |  |  |
| 518 | 04-Mar-22 | Omicron BA.2 | Yes | Possible Omicron Stealth (BA.2) |  |  | Omicron (BA.2) | EPI_ISL_11609752 |
| 519 | 07-Mar-22 | Omicron BA.2 | No | Non-omicron/ possible Delta |  |  | Omicron (BA.2) | EPI_ISL_11609753 |
| 520 | 07-Mar-22 | Omicron BA.1 |  |  | Omicron V2 panel | Omicron (BA.1) |  |  |
| 521 | 07-Mar-22 | Omicron BA.1 |  |  | Omicron V2 panel | Omicron (BA.1) |  |  |
| 522 | 05-Mar-22 | Omicron BA.2 |  |  | Omicron V2 panel | Omicron (BA.2) |  |  |
| 523 | 05-Mar-22 | Omicron BA.2 |  |  | Omicron V2 panel | Omicron (BA.2) |  |  |
| 524 | 05-Mar-22 | Omicron BA.2 |  |  | Omicron V2 panel | Omicron (BA.2) |  |  |
| 525 | 05-Mar-22 | Omicron BA.2 |  |  | Omicron V2 panel | Omicron (BA.2) |  |  |
| 526 | 05-Mar-22 | Omicron BA.1 |  |  | Omicron V2 panel | Omicron (BA.1) |  |  |
| 527 | 05-Mar-22 | Omicron BA.2 |  |  | Omicron V2 panel | Omicron (BA.2) |  |  |
| 528 | 05-Mar-22 | Omicron BA.2 |  |  | Omicron V2 panel | Omicron (BA.2) |  |  |
| 529 | 05-Mar-22 | Omicron BA.2 |  |  | Omicron V2 panel | Omicron (BA.2) |  |  |
| 530 | 05-Mar-22 | Omicron BA.1 |  |  | Omicron V2 panel | Omicron (BA.1) |  |  |
| 531 | 05-Mar-22 | Omicron BA.1 |  |  | Omicron V2 panel | Omicron (BA.1) |  |  |
| 532 | 05-Mar-22 | Omicron BA.2 |  |  | Omicron V2 panel | Omicron (BA.2) |  |  |
| 533 | 05-Mar-22 | Omicron BA.1 |  |  | Omicron V2 panel | Omicron (BA.1) |  |  |
| 534 | 05-Mar-22 | Omicron BA.2 |  |  | Omicron V2 panel | Omicron (BA.2) |  |  |
| 535 | 05-Mar-22 | Omicron BA.2 |  |  | Omicron V2 panel | Omicron (BA.2) |  |  |
| 536 | 05-Mar-22 | Omicron BA.2 |  |  | Omicron V2 panel | Omicron (BA.2) |  |  |
| 537 | 05-Mar-22 | Omicron BA.2 |  |  | Omicron V2 panel | Omicron (BA.2) |  |  |
| 538 | 05-Mar-22 | Omicron BA.1 |  |  | Omicron V2 panel | Omicron (BA.1) |  |  |
| 539 | 05-Mar-22 | Omicron BA.2 |  |  | Omicron V2 panel | Omicron (BA.2) |  |  |
| 540 | 05-Mar-22 | Omicron BA.1 |  |  | Omicron V2 panel | Omicron (BA.1) |  |  |
| 541 | 05-Mar-22 | Omicron BA.2 |  |  | Omicron V2 panel | Omicron (BA.2) |  |  |
| 542 | 06-Mar-22 | Omicron BA.2 |  |  | Omicron V2 panel | Omicron (BA.2) |  |  |
| 543 | 06-Mar-22 | Omicron BA.1 |  |  | Omicron V2 panel | Omicron (BA.1) |  |  |
| 544 | 06-Mar-22 | Omicron BA.1 |  |  | Omicron V2 panel | Omicron (BA.1) |  |  |
| 545 | 06-Mar-22 | Omicron BA.2 |  |  | Omicron V2 panel | Omicron (BA.2) |  |  |
| 546 | 06-Mar-22 | Omicron BA.2 |  |  | Omicron V2 panel | Omicron (BA.2) |  |  |
| 547 | 06-Mar-22 | Omicron BA.2 |  |  | Omicron V2 panel | Omicron (BA.2) |  |  |
| 548 | 06-Mar-22 | Omicron BA.2 |  |  | Omicron V2 panel | Omicron (BA.2) |  |  |
| 549 | 06-Mar-22 | Omicron BA.2 |  |  | Omicron V2 panel | Omicron (BA.2) |  |  |
| 550 | 06-Mar-22 | Omicron BA.2 |  |  | Omicron V2 panel | Omicron (BA.2) |  |  |
| 551 | 06-Mar-22 | Omicron BA.1 |  |  | Omicron V2 panel | Omicron (BA.1) |  |  |
| 552 | 06-Mar-22 | Omicron BA.2 |  |  | Omicron V2 panel | Omicron (BA.2) |  |  |
| 553 | 06-Mar-22 | Omicron BA.2 |  |  | Omicron V2 panel | Omicron (BA.2) |  |  |
| 554 | 06-Mar-22 | Omicron BA.1 |  |  | Omicron V2 panel | Omicron (BA.1) |  |  |
| 555 | 07-Mar-22 | Omicron BA.2 |  |  | Omicron V2 panel | Omicron (BA.2) |  |  |
| 556 | 07-Mar-22 | Omicron BA.1 |  |  | Omicron V2 panel | Omicron (BA.1) |  |  |
| 557 | 07-Mar-22 | Failed |  | Unidentified |  |  |  |  |
| 558 | 07-Mar-22 | Omicron BA.2 | Yes | Possible Omicron Stealth (BA.2) |  |  | Omicron (BA.2) | EPI_ISL_11609771 |
| 559 | 07-Mar-22 | Omicron BA.2 |  | Possible Omicron Stealth (BA.2) |  |  |  |  |
| 560 | 07-Mar-22 | Omicron BA.2 | Yes | Possible Omicron Stealth (BA.2) |  |  | Omicron (BA.2) | EPI_ISL_11609754 |
| 561 | 07-Mar-22 | Omicron BA.2 |  | Possible Omicron Stealth (BA.2) |  |  |  |  |
| 562 | 07-Mar-22 | Omicron BA.2 |  | Possible Omicron Stealth (BA.2) |  |  |  |  |
| 563 | 07-Mar-22 | Omicron BA.2 |  | Possible Omicron Stealth (BA.2) |  |  |  |  |
| 564 | 08-Mar-22 | Omicron BA.1 |  | Possible Omicron (B.1.1.529) |  |  |  |  |
| 565 | 11-Mar-22 | Omicron BA.2 | Yes | Possible Omicron Stealth (BA.2) |  |  | Omicron (BA.2) | EPI_ISL_11609769 |
| 566 | 11-Mar-22 | Omicron BA.1 |  | Possible Omicron (B.1.1.529) |  |  |  |  |
| 567 | 11-Mar-22 | Omicron BA.1 | Yes | Possible Omicron (B.1.1.529) |  |  | Omicron (BA.1.1) | EPI_ISL_11609762 |
| 568 | 11-Mar-22 | Omicron BA.2 | Yes | Possible Omicron Stealth (BA.2) |  |  | Omicron (BA.2) | EPI_ISL_11609755 |
| 569 | 14-Mar-22 | Failed |  | Unidentified |  |  |  |  |
| 570 | 14-Mar-22 | Omicron BA.2 | Yes | Possible Omicron Stealth (BA.2) | Omicron V2 panel | Omicron (BA.2) |  |  |
| 571 | 14-Mar-22 | Omicron BA.2 | Yes | Possible Omicron Stealth (BA.2) |  |  | Omicron (BA.2) | EPI_ISL_11609756 |
| 572 | 15-Mar-22 | Omicron BA.2 |  | Possible Omicron Stealth (BA.2) |  |  |  |  |
| 573 | 15-Mar-22 | Omicron BA.2 |  | Possible Omicron Stealth (BA.2) |  |  |  |  |
| 574 | 14-Mar-22 | Omicron BA.2 |  | Possible Omicron Stealth (BA.2) |  |  |  |  |
| 575 | 14-Mar-22 | Omicron BA.2 | Yes | Possible Omicron Stealth (BA.2) |  |  | Omicron (BA.2) | EPI_ISL_11609757 |
| 576 | 14-Mar-22 | Omicron BA.2 |  | Possible Omicron Stealth (BA.2) |  |  |  |  |
| 577 | 14-Mar-22 | Omicron BA.1 | No | Non-omicron/ possible Delta | Omicron V2 panel | Omicron (BA.1) | Failed |  |
| 578 | 15-Mar-22 | Omicron BA.2 | Yes | Possible Omicron Stealth (BA.2) |  |  | Omicron (BA.2) | EPI_ISL_11609758 |
| 579 | 15-Mar-22 | Omicron BA.2 | Yes | Possible Omicron Stealth (BA.2) |  |  | Omicron (BA.2) | EPI_ISL_11609759 |
| 580 | 16-Mar-22 | Omicron BA.1 |  | Possible Omicron (B.1.1.529) |  |  |  |  |
| 581 | 16-Mar-22 | Omicron BA.1 | Yes | Possible Omicron (B.1.1.529) |  |  | Omicron (BA.1.1) | EPI_ISL_11610488 |
| 582 | 16-Mar-22 | Omicron BA.2 | Yes | Possible Omicron Stealth (BA.2) |  |  | Omicron (BA.2) | EPI_ISL_11609760 |
| 583 | 16-Mar-22 | Omicron BA.2 |  | Possible Omicron Stealth (BA.2) |  |  |  |  |
| 584 | 16-Mar-22 | Omicron BA.2 |  | Possible Omicron Stealth (BA.2) |  |  |  |  |
| 585 | 15-Mar-22 | Failed |  |  |  |  | Failed |  |
| 586 | 15-Mar-22 | Failed |  |  |  |  | Failed |  |
| 587 | 17-Mar-22 | Omicron BA.2 |  | Possible Omicron Stealth (BA.2) |  |  |  |  |
| 588 | 18-Mar-22 | Omicron BA.2 |  | Possible Omicron Stealth (BA.2) |  |  |  |  |
| 589 | 18-Mar-22 | Omicron BA.1 |  | Possible Omicron (B.1.1.529) |  |  |  |  |
| 590 | 19-Mar-22 | Omicron BA.2 |  | Possible Omicron Stealth (BA.2) |  |  |  |  |
| 591 | 19-Mar-22 | Omicron BA.2 |  | Possible Omicron Stealth (BA.2) |  |  |  |  |
| 592 | 19-Mar-22 | Omicron BA.2 |  | Possible Omicron Stealth (BA.2) |  |  |  |  |
| 593 | 19-Mar-22 | Omicron BA.2 |  | Possible Omicron Stealth (BA.2) |  |  |  |  |
| 594 | 20-Mar-22 | Omicron BA.2 |  | Possible Omicron Stealth (BA.2) |  |  |  |  |
| 595 | 20-Mar-22 | Omicron BA.1 |  | Possible Omicron (B.1.1.529) |  |  |  |  |
| 596 | 20-Mar-22 | Omicron BA.2 |  | Possible Omicron Stealth (BA.2) |  |  |  |  |
| 597 | 20-Mar-22 | Omicron BA.2 |  | Possible Omicron Stealth (BA.2) |  |  |  |  |
| 598 | 20-Mar-22 | Omicron BA.2 |  | Possible Omicron Stealth (BA.2) |  |  |  |  |
| 599 | 20-Mar-22 | Omicron BA.2 |  | Possible Omicron Stealth (BA.2) |  |  |  |  |
| 600 | 20-Mar-22 | Omicron BA.2 |  | Possible Omicron Stealth (BA.2) |  |  |  |  |
| 601 | 20-Mar-22 | Omicron BA.2 |  | Possible Omicron Stealth (BA.2) |  |  |  |  |
| 602 | 20-Mar-22 | Omicron BA.2 |  | Possible Omicron Stealth (BA.2) |  |  |  |  |
| 603 | 20-Mar-22 | Omicron BA.2 |  | Possible Omicron Stealth (BA.2) |  |  |  |  |
| 604 | 21-Mar-22 | Omicron BA.1 |  | Possible Omicron (B.1.1.529) |  |  |  |  |
| 605 | 22-Mar-22 | Omicron BA.1 |  | Possible Omicron (B.1.1.529) |  |  |  |  |
| 606 | 22-Mar-22 | Omicron BA.2 |  | Possible Omicron Stealth (BA.2) |  |  |  |  |
| 607 | 22-Mar-22 | Omicron BA.2 |  | Possible Omicron Stealth (BA.2) |  |  |  |  |
| 608 | 22-Mar-22 | Omicron BA.2 |  | Possible Omicron Stealth (BA.2) |  |  |  |  |
| 609 | 24-Mar-22 | Omicron BA.2 |  | Possible Omicron Stealth (BA.2) |  |  |  |  |
| 610 | 24-Mar-22 | Omicron BA.2 |  | Possible Omicron Stealth (BA.2) |  |  |  |  |
| 611 | 24-Mar-22 | Omicron BA.2 |  | Possible Omicron Stealth (BA.2) |  |  |  |  |
| 612 | 28-Mar-22 | Omicron BA.1 | Yes | Possible Omicron (B.1.1.529) | Omicron V2 panel | Omicron (BA.1) |  |  |
| 613 | 27-Mar-22 | Omicron BA.2 | Yes | Possible Omicron Stealth (BA.2) | Omicron V3 panel | Omicron (BA.2) |  |  |
| 614 | 29-Mar-22 | Omicron BA.2 |  | Possible Omicron Stealth (BA.2) |  |  |  |  |
| 615 | 28-Mar-22 | Omicron BA.2 |  | Possible Omicron Stealth (BA.2) |  |  |  |  |
| 616 | 29-Mar-22 | Omicron BA.2 | Yes | Possible Omicron Stealth (BA.2) | Omicron V3 panel | Omicron (BA.2) |  |  |
| 617 | 29-Mar-22 | Omicron BA.1 | No | Possible Omicron Stealth (BA.2) | Omicron V3 panel | Omicron (BA.1) |  |  |
| 618 | 30-Mar-22 | Omicron BA.1 | No | Possible Omicron Stealth (BA.2) | Omicron V3 panel | Omicron (BA.1) |  |  |

Table S2. New daily SARS-CoV-2 cases in Bangkok and Thailand were retrieved from the Thailand Department of Disease Control COVID-19 API (https://ddc.moph.go.th/covid19-daily-dashboard/).

| **Date** | **Thailand new cases/day** | **Bangkok new cases/day** |
| --- | --- | --- |
| 1-Nov-21 | 8165 | 701 |
| 2-Nov-21 | 7574 | 823 |
| 3-Nov-21 | 7679 | 807 |
| 4-Nov-21 | 7980 | 872 |
| 5-Nov-21 | 8150 | 741 |
| 6-Nov-21 | 8467 | 923 |
| 7-Nov-21 | 7960 | 765 |
| 8-Nov-21 | 7592 | 879 |
| 9-Nov-21 | 6904 | 829 |
| 10-Nov-21 | 6978 | 957 |
| 11-Nov-21 | 7496 | 761 |
| 12-Nov-21 | 7305 | 684 |
| 13-Nov-21 | 7057 | 698 |
| 14-Nov-21 | 7079 | 712 |
| 15-Nov-21 | 6343 | 794 |
| 16-Nov-21 | 5947 | 859 |
| 17-Nov-21 | 6524 | 931 |
| 18-Nov-21 | 6901 | 910 |
| 19-Nov-21 | 6855 | 811 |
| 20-Nov-21 | 6595 | 842 |
| 21-Nov-21 | 7006 | 780 |
| 22-Nov-21 | 6428 | 832 |
| 23-Nov-21 | 5126 | 775 |
| 24-Nov-21 | 5857 | 760 |
| 25-Nov-21 | 6335 | 676 |
| 26-Nov-21 | 6559 | 623 |
| 27-Nov-21 | 6073 | 722 |
| 28-Nov-21 | 5854 | 667 |
| 29-Nov-21 | 4753 | 606 |
| 30-Nov-21 | 4306 | 744 |
| 1-Dec-21 | 4886 | 797 |
| 2-Dec-21 | 4971 | 786 |
| 3-Dec-21 | 4912 | 699 |
| 4-Dec-21 | 5896 | 943 |
| 5-Dec-21 | 4704 | 795 |
| 6-Dec-21 | 4000 | 784 |
| 7-Dec-21 | 3525 | 818 |
| 8-Dec-21 | 3618 | 853 |
| 9-Dec-21 | 4203 | 861 |
| 10-Dec-21 | 4193 | 647 |
| 11-Dec-21 | 4079 | 672 |
| 12-Dec-21 | 3787 | 641 |
| 13-Dec-21 | 3398 | 608 |
| 14-Dec-21 | 2862 | 589 |
| 15-Dec-21 | 3370 | 637 |
| 16-Dec-21 | 3684 | 604 |
| 17-Dec-21 | 3537 | 588 |
| 18-Dec-21 | 3132 | 589 |
| 19-Dec-21 | 2899 | 430 |
| 20-Dec-21 | 2525 | 420 |
| 21-Dec-21 | 2476 | 578 |
| 22-Dec-21 | 2532 | 444 |
| 23-Dec-21 | 2940 | 472 |
| 24-Dec-21 | 2671 | 447 |
| 25-Dec-21 | 2766 | 505 |
| 26-Dec-21 | 2532 | 436 |
| 27-Dec-21 | 2437 | 453 |
| 28-Dec-21 | 2305 | 433 |
| 29-Dec-21 | 2575 | 407 |
| 30-Dec-21 | 3037 | 496 |
| 31-Dec-21 | 3111 | 460 |
| 1-Jan-22 | 3011 | 454 |
| 2-Jan-22 | 3112 | 408 |
| 3-Jan-22 | 2927 | 395 |
| 4-Jan-22 | 3091 | 388 |
| 5-Jan-22 | 3899 | 436 |
| 6-Jan-22 | 5775 | 496 |
| 7-Jan-22 | 7526 | 493 |
| 8-Jan-22 | 8263 | 713 |
| 9-Jan-22 | 8511 | 614 |
| 10-Jan-22 | 7926 | 561 |
| 11-Jan-22 | 7133 | 640 |
| 12-Jan-22 | 7681 | 939 |
| 13-Jan-22 | 8167 | 857 |
| 14-Jan-22 | 8158 | 799 |
| 15-Jan-22 | 7793 | 839 |
| 16-Jan-22 | 8077 | 829 |
| 17-Jan-22 | 6929 | 760 |
| 18-Jan-22 | 6397 | 782 |
| 19-Jan-22 | 7122 | 843 |
| 20-Jan-22 | 8129 | 1214 |
| 21-Jan-22 | 8640 | 1667 |
| 22-Jan-22 | 8112 | 1167 |
| 23-Jan-22 | 7686 | 1182 |
| 24-Jan-22 | 7139 | 1157 |
| 25-Jan-22 | 6718 | 1279 |
| 26-Jan-22 | 7587 | 1701 |
| 27-Jan-22 | 8078 | 1446 |
| 28-Jan-22 | 8450 | 1323 |
| 29-Jan-22 | 8618 | 1520 |
| 30-Jan-22 | 8444 | 1280 |
| 31-Jan-22 | 8008 | 1316 |
| 1-Feb-22 | 7422 | 1269 |
| 2-Feb-22 | 8587 | 1439 |
| 3-Feb-22 | 9172 | 1458 |
| 4-Feb-22 | 9909 | 1619 |
| 5-Feb-22 | 10490 | 1521 |
| 6-Feb-22 | 10879 | 1485 |
| 7-Feb-22 | 10470 | 1407 |
| 8-Feb-22 | 10398 | 1900 |
| 9-Feb-22 | 13182 | 2772 |
| 10-Feb-22 | 14822 | 2670 |
| 11-Feb-22 | 15242 | 3052 |
| 12-Feb-22 | 16330 | 3244 |
| 13-Feb-22 | 15882 | 2962 |
| 14-Feb-22 | 14900 | 2915 |
| 15-Feb-22 | 14373 | 3184 |
| 16-Feb-22 | 16462 | 2950 |
| 17-Feb-22 | 17349 | 3083 |
| 18-Feb-22 | 18066 | 3518 |
| 19-Feb-22 | 18885 | 3226 |
| 20-Feb-22 | 18953 | 2755 |
| 21-Feb-22 | 18883 | 2761 |
| 22-Feb-22 | 18363 | 2650 |
| 23-Feb-22 | 21232 | 3165 |
| 24-Feb-22 | 23557 | 3266 |
| 25-Feb-22 | 24932 | 3346 |
| 26-Feb-22 | 25615 | 3270 |
| 27-Feb-22 | 24719 | 2678 |
| 28-Feb-22 | 22311 | 2781 |
| 1-Mar-22 | 20420 | 2802 |
| 2-Mar-22 | 22197 | 3054 |
| 3-Mar-22 | 23618 | 2810 |
| 4-Mar-22 | 23834 | 2620 |
| 5-Mar-22 | 22818 | 2836 |
| 6-Mar-22 | 21881 | 2184 |
| 7-Mar-22 | 21162 | 2815 |
| 8-Mar-22 | 18943 | 2949 |
| 9-Mar-22 | 22073 | 3190 |
| 10-Mar-22 | 22984 | 3216 |
| 11-Mar-22 | 24792 | 3202 |
| 12-Mar-22 | 24592 | 3009 |
| 13-Mar-22 | 23584 | 2982 |
| 14-Mar-22 | 22130 | 3060 |
| 15-Mar-22 | 19742 | 2913 |
| 16-Mar-22 | 23945 | 3473 |
| 17-Mar-22 | 25456 | 3707 |
| 18-Mar-22 | 27071 | 3273 |
| 19-Mar-22 | 25804 | 3341 |
| 20-Mar-22 | 24996 | 2925 |
| 21-Mar-22 | 23441 | 2872 |
| 22-Mar-22 | 21382 | 3112 |
| 23-Mar-22 | 25164 | 3387 |
| 24-Mar-22 | 27024 | 3732 |
| 25-Mar-22 | 26050 | 3741 |
| 26-Mar-22 | 25821 | 3136 |
| 27-Mar-22 | 24635 | 2792 |
| 28-Mar-22 | 21678 | 3250 |
| 29-Mar-22 | 25389 | 3637 |
| 30-Mar-22 | 24560 | 3348 |
| 31-Mar-22 | 28379 | 3378 |
